# A spatial simulation model for dengue virus infection in urban areas

**DOI:** 10.1186/1471-2334-14-447

**Published:** 2014-08-20

**Authors:** Stephan Karl, Nilimesh Halder, Joel K Kelso, Scott A Ritchie, George J Milne

**Affiliations:** School of Computer Science and Software Engineering, The University of Western Australia, 35 Stirling Highway, Crawley, Perth, WA 6009 Australia; School of Public Health, Tropical Medicine & Rehabilitation Sciences, James Cook University, Cairns, Queensland Australia

**Keywords:** Dengue virus, Individual-based simulation model, *Ae. aegypti* population dynamics, Spatially-explicit modelling, Dengue outbreaks, Dengue control measures

## Abstract

**Background:**

The World Health Organization estimates that the global number of dengue infections range between 80–100 million per year, with some studies estimating approximately three times higher numbers. Furthermore, the geographic range of dengue virus transmission is extending with the disease now occurring more frequently in areas such as southern Europe. *Ae. aegypti*, one of the most prominent dengue vectors, is endemic to the far north-east of Australia and the city of Cairns frequently experiences dengue outbreaks which sometimes lead to large epidemics.

**Method:**

A spatially-explicit, individual-based mathematical model that accounts for the spread of dengue infection as a result of human movement and mosquito dispersion is presented. The model closely couples the four key sub-models necessary for representing the overall dynamics of the physical system, namely those describing mosquito population dynamics, human movement, virus transmission and vector control. Important features are the use of high quality outbreak data and mosquito trapping data for calibration and validation and a strategy to derive local mosquito abundance based on vegetation coverage and census data.

**Results:**

The model has been calibrated using detailed 2003 dengue outbreak data from Cairns, together with census and mosquito trapping data, and is shown to realistically reproduce a further dengue outbreak. The simulation results replicating the 2008/2009 Cairns epidemic support several hypotheses (formulated previously) aimed at explaining the large-scale epidemic which occurred in 2008/2009; specifically, while warmer weather and increased human movement had only a small effect on the spread of the virus, a shorter virus strain-specific extrinsic incubation time can explain the observed explosive outbreak of 2008/2009.

**Conclusion:**

The proof-of-concept simulation model described in this study has potential as a tool for understanding factors contributing to dengue spread as well as planning and optimizing dengue control, including reducing the *Ae. aegypti* vector population and for estimating the effectiveness and cost-effectiveness of future vaccination programmes. This model could also be applied to other vector borne viral diseases such as chikungunya, also spread by *Ae. aegypti* and, by re-parameterisation of the vector sub-model, to dengue and chikungunya viruses spread by *Aedes albopictus.*

**Electronic supplementary material:**

The online version of this article (doi:10.1186/1471-2334-14-447) contains supplementary material, which is available to authorized users.

## Background

Dengue is a leading cause of morbidity in tropical environments around the world [1] with a small proportion of infections resulting in possibly fatal dengue hemorrhagic fever (DHF) [2]. While previous estimates of the global dengue burden were in the range of 80–100 million human infections per year, recent studies suggest a considerably higher number of infections (390 million per year) [3]. There are also clear indications that the global range of dengue transmission is extending (e.g. in Southern Europe), with higher case numbers occurring and *Aedes* mosquitoes colonizing new habitats [4, 5].

Dengue epidemics can be especially severe in urban areas, where human population density is high [6]. The vector mosquito *Ae. aegypti* have adapted to life in densely populated areas, using standing water as breeding sites with females feeding predominantly on humans, and are responsible for dengue transmission in urban environments [7].

Controlling dengue outbreaks is resource intensive and large outbreaks may overwhelm even well established and efficient control mechanisms [7]. It is therefore important for the planning of adequate control interventions that the dynamics, frequency and scale of expected outbreaks can be predicted and simulated using suitable computational models [8]. Furthermore, such simulation models can be used to estimate the effectiveness of alternative mitigating intervention strategies that are difficult to determine in the field [9].

*Ae. aegypti* is endemic to urban areas of northeast Queensland, Australia. Dengue viruses are often introduced to this region through viremic travellers from dengue endemic regions. This frequently causes dengue outbreaks of variable severity and the city of Cairns is often the focus of these outbreaks [7, 10].

Long-term dengue research and surveillance, including mosquito trapping studies in Cairns, has resulted in the collation of significant datasets describing dengue outbreaks in this area [7, 10–13]. These datasets were made available to the authors from the Tropical Population Health Unit (TPHU) of Queensland Health. These data have allowed for the informed development of a detailed dengue simulation model which may be used to estimate and evaluate the characteristics and determinants of dengue outbreaks and to test a range of interventions such as interior (within-house) residual spraying (IRS), the larvicidal treatment or destruction of *Aedes* breeding sites, potential dengue vaccination programmes and mosquito population manipulation using *Wolbachia* infected mosquitoes [14].

The aim of the present study was to develop a proof-of-concept, spatially-explicit simulation model of dengue transmission that incorporates individual humans living in the city of Cairns as well as individual mosquitoes, and uses realistic, heterogeneous human and mosquito population structures and movement patterns. A spatial modelling approach is required since many of the factors that are crucial for dengue spread are not homogeneously distributed (e.g., human population density and mosquito abundance).

The model developed in the present study builds upon previous studies which have modelled mosquito population dynamics and/or dengue transmission. The model presented here adopts many of the features of the previously developed CIMSIM/DENSIM and Skeeterbuster models, which are highly developed, complex simulation models that are focused on capturing mosquito population dynamics with great detail using a complex system of customizable mosquito breeding containers and weather data as input [15–25].

The motivation of the present study was *i)* to build a spatial mosquito population dynamics sub-model which is weather mediated and results in physically realistic temporal and spatial mosquito abundance patterns; *ii)* to build human population, dengue transmission and outbreak management sub-models with internal feedback (that is, dengue control has an effect on the mosquito population) and couple this to the mosquito population sub-model and *iii)* to calibrate and validate the model using detailed data available for several dengue outbreaks in the city of Cairns, Queensland.

The resulting model structure utilises new modelling techniques to capture physical system properties such as mosquito flight, rainfall dependence of the mosquito population, human movement patterns and dengue control. Here, the model is demonstrated by reproducing different outbreak scenarios recorded in Cairns. This is one of very few spatial dengue transmission model that include all of the following features *i)* human movement and mosquito flight, *ii)* geospatial estimation of mosquito breeding site abundance in an urban area, *iii)* internal feedback of individual-based mosquito control during a dengue outbreak on the mosquito population dynamics and *iv)* the use of detailed, spatial outbreak datasets to calibrate and, separately, validate the model.

The developed model is a *proof-of-concept demonstration* that the inherently complex phenomena observed in actual dengue outbreaks can be captured by a spatial simulation model that incorporates interlinked and interacting sub-models for each of the phenomena that determine the outcome of dengue epidemics. These are: mosquito population dynamics and movement; human population movement; transmission of dengue virus between mosquitos and humans; and vector control measures.

While the following methods section illustrates the overall approach to building, calibrating and validating the model, a detailed description of the various model components may be found in Additional file 1.

## Methods

### Modelling strategy

**Figure 1:**
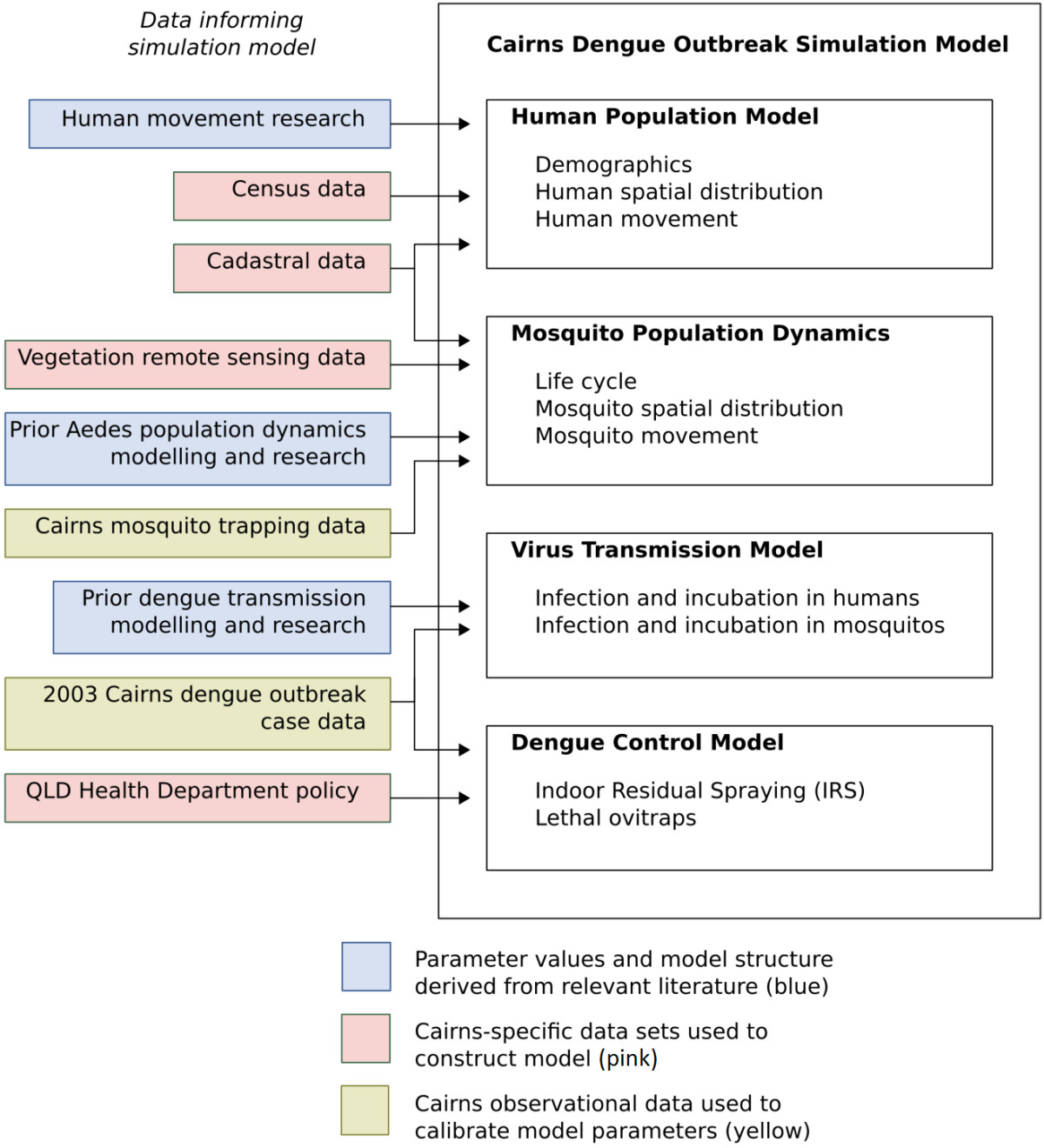
**Data informing the dengue simulation model.** A variety of data sources were used to build the dengue simulation model consisting of 4 key components i) a human population sub-model, ii) a mosquito population sub-model, iii) a dengue virus transmission sub- model and iv) a dengue control sub-model. It should be noted that a mosquito population dynamics sub-model similar to that previously described by Otero et al. [23, 25] was used, with modifications as described in the Additional file 1.

**Figure 2:**
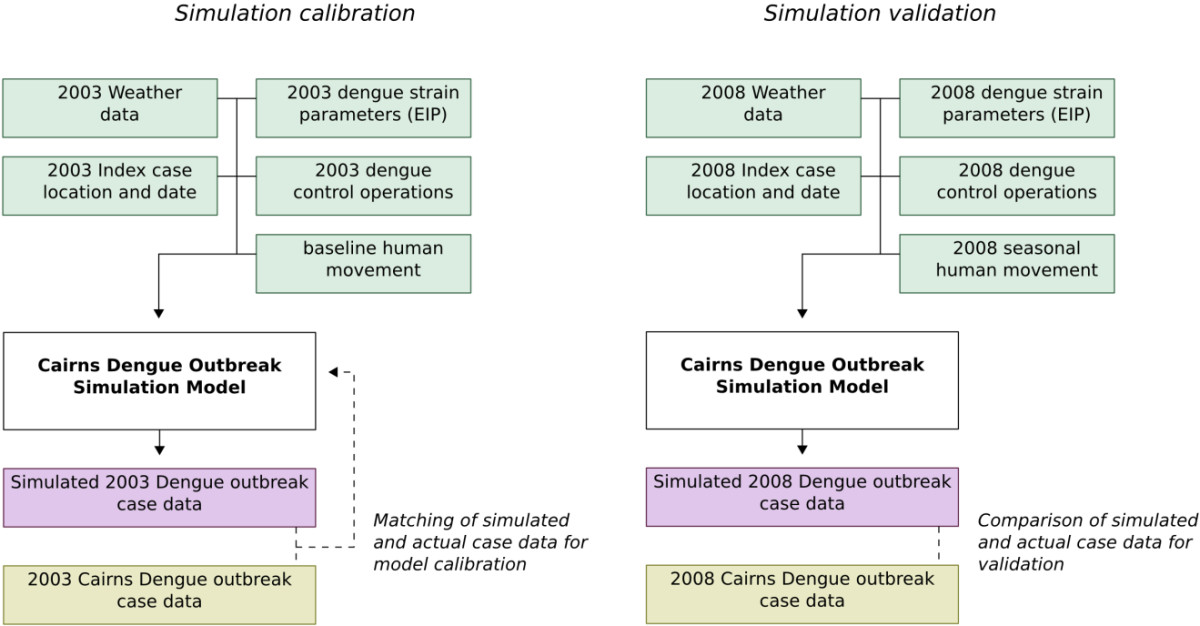
**Schematic outline of strategy applied to calibrate and validate the dengue simulation model.** Detailed data from a 2003 dengue outbreak in Cairns were used to calibrate the model by adjustment of uncertain parameters. Detailed data from a 2008/2009 epidemic were used to validate the resulting model.

The approach adopted utilised as much field data as possible to inform model development, calibration and, subsequently, validation. The model consists of the following 4 linked sub-model components representing: *i)* mosquito population dynamics and movement, *ii)* human population movement, *iii)* dengue virus transmission and *iv)* dengue control. Each of these component parts are described in the detailed Additional file 1. In this section we focus on key approaches adopted for the design, calibration and implementation of each part of the model.Figure 1 shows a schematic of the different sets of data that inform each of the component submodels of the complete dengue transmission model.Detailed outbreak data from Cairns was available for two dengue outbreaks (2003 and 2008/2009), hence one of the datasets (2003) was used to calibrate the model and the other dataset (2008/2009) to validate the overall model, including parameter choices arising from the calibration procedure. The following schematic (Figure 2) shows the overall model and the strategy used for model calibration and validation.

Figure 2 indicates how four types of input data were used, and these data differed between the 2003 and the 2008/2009 outbreaks: *i)* weather data, *ii)* dengue strain specific extrinsic incubation period data, *iii)* index case location data and *iv)* data describing the dengue control operations undertaken in the two outbreaks.

### Model description

The overall methods applied in this study are presented in the following. A detailed desciption of all model components is given in Additional file 1.

**Figure 3:**
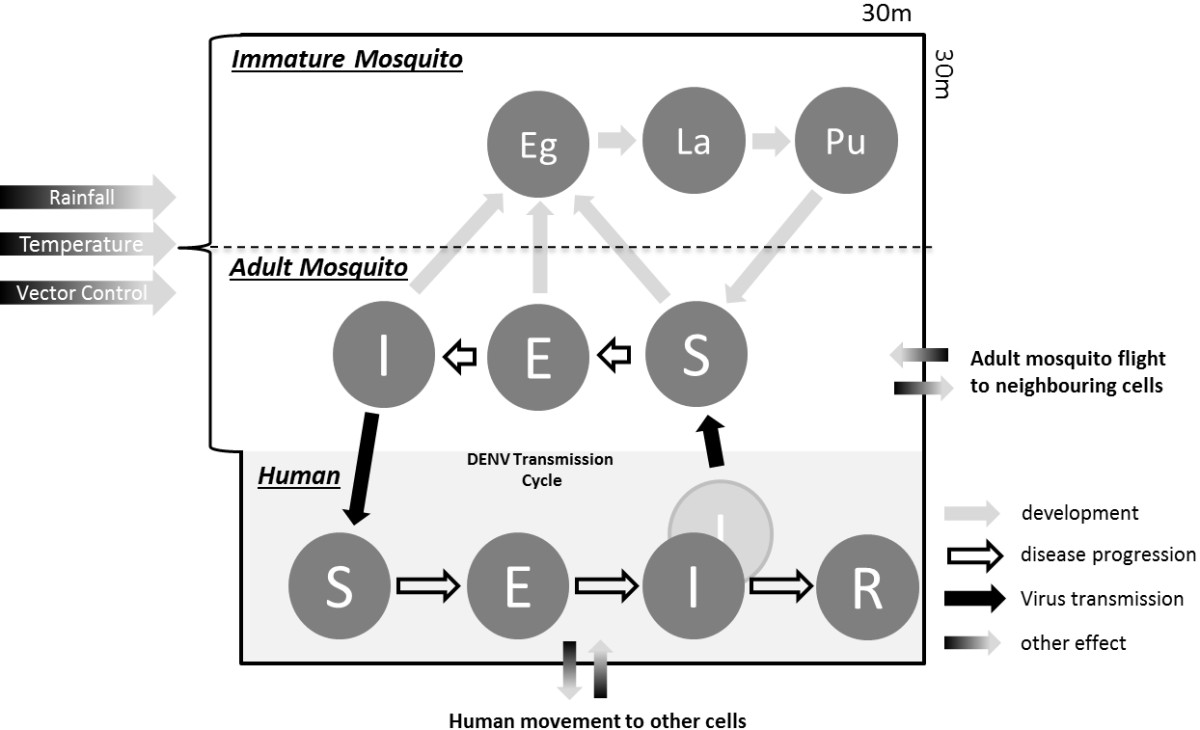
**Schematic representation of a single model cell.** Mosquito population dynamics, human movement, infection dynamics and mosquito flight characteristics are calculated for each cell. Mosquito population dynamics are governed by the weather, vector control and mosquito flight characterisitcs. Human movement patterns are subject to the time and type of day (morning, daytime, evening, night, weekday, weekend). In the human disease progression, the dark ‘I’ indicates symptomatic infections and the grey ‘I’ indicates asymptomatic infections (a detailed explanation of symptomatic and asymptomatic infections is presented in the Additional file 1: Figure S24).

A discrete event simulation approach was adopted with both space and time being represented discretely [26]. All processes occuring in the basic spatial unit of the model (called a *cell*) are shown in Figure 3. Each model cell is a 30 × 30 m square and is thus smaller than the maximum known adult mosquito flight distance (usually limited to 200 m around the location of adult mosquito emergence, so permitting localised vector infection to be represented following introduction of an infectious case into that cell location [13]. This cell size corresponds to an area containing (on average) 1 to 3 households in a Cairns setting.

### Human population model

The human population part of the model is based on previous models designed by Milne and colleagues but with important differences e.g., in human movement behaviour as detailed below and in the Additional file 1 (pages S2-S7) [27–30].

The model of Cairns consists of approximately 52,000 cells (Additional file 1: Figures S2 and S4) and 56,000 human individuals. To assign a human population to the cells, two sets of data were used: *i)* the most recent population census from the Australian Bureau of Statistics (accessible at http://www.abs.gov.au, illustrated in Additional file 1: Figures S2 and S3) and *ii)* a geo-referenced digital cadastral dataset from Cairns provided by the Department of Natural Resources and Mines of the Queensland Government (accessible at http://www.qld.gov.au, illustrated in Additional file 1: Figures S2 - S4).

While the census data provided information on human population age distribution (illustrated in Additional file 1: Figure S1 for all of Cairns), household sizes and the number of people per area at a high spatial resolution (Additional file 1: Figure S2 Panels A and B), the cadastral dataset specified *residential*, *commercial*, *industrial*, *educational* and *parkland* associated properties (Additional file 1: Figure S2 Panels C and D). This made it possible to allocate households of appropriate sizes and with appropriate age distributions to each residential property. Thus, each human is assigned a home cell. Apart from their home cells, and depending on their age, humans may also frequent other cells. Properties classified as commercial and industrial in the cadastral dataset served as workplaces and shopping centres that are frequented by the human population. Data on schools (student numbers, class sizes) were sourced from the Queensland school register (accessible at http://www.education.qld.gov.au) for schools in the modelled area. Appropriate virtual classes of school children, including their teachers were built and associated with the educational establishments listed in the cadastral dataset. In addition, parkland areas are also frequented by humans for recreational purposes. A single cell in the model can contain more than one property and also properties of different types (e.g. Additional file 1: Figure S4 Panel B). We assume no further boundaries between all humans allocated to a single cell (e.g., mosquito access to humans allocated to two separate households which are located in the same cell will be the same).

In the model, time progresses in a step-wise (discrete) manner and in intervals of 6 hours, i.e. infection states and locations of humans and mosquitoes *may* change every 6 hours. Each 6 hour period corresponds with a specific part of the day: *i)* morning (3 am to 9 am), *ii)* daytime (9 am to 3 pm), *iii)* evening (3 pm to 9 pm) and *iv)* night (9 pm to 3 am). Humans are assumed to move between cells during the daytime and evening periods, and are assumed to be in their home cells during morning and night periods.

It has been shown that human movement is an important factor facilitating the spread of dengue [31]. Detailed information on human movement is very sparse; we used the findings of a large survey of interpersonal human contact to derive an approximate model of daily movement of individuals, which apportions each person’s time between different locations [32]. This model incorporates two types of human movement.

The first type is directional and always occurs between two specific cells for a given individual. These cells are *i)* the individual’s home cell and *ii)* the individual’s work or school cell. On any given weekday of the modelled period, individuals will go from their home cells (morning) to their work or school cells (daytime) and back to their home cells (evening or night), following the approach adopted in Milne et al. [27].

The second type of human movement used in the present study is semi-random and is incorporated in the model to account for the less predictable movement which humans may undertake within the modelled area e.g., to visit friends, go shopping, or visit recreation areas. This semi-random movement can occur on weekday evenings and during the day and evening during weekends. Based on data from a population survey by Mossong et al. it was estimated that the frequency of random human visits of this type to other cells is approximately 4–5 times per week per individual [32]. A schematic representation of the human movement components of the present model is shown in Additional file 1: Figure S5.

Human movement in urban areas has been shown to be semi-random and to be ranked by distance i.e., the frequency of short trips is higher than the frequency of trips to destinations that are further away [9]. In the model, this distance-dependent behaviour is modelled with a *gamma* distribution of the semi-random movement distances (Additional file 1: Figure S6) [27].

### Mosquito population dynamics model

The mosquito population dynamics model used in the present study is based on previously developed models by others [17, 23, 25, 33]. The core mosquito population dynamics model (only female adult mosquitoes) is shown in Additional file 1: Figure S7. It includes egg (E), larvae (L), pupae (P) and two adult mosquito populations (A1 and A2) as determined by the length of the first (A1) versus the lengths of the remaining (A2) gonotrophic cycles, with the first gonotrophic cycle being significantly longer than the remaining ones [16].

The mosquito population sub-model is similar to that described in previous studies but it also differs from these in a number of ways, such as details in the implementation of larvae density dependent parameters and mosquito flight [17, 23, 24, 33]. Specific details of the mosquito population dynamics model are given in the Additional file 1 (pages S8-S23 with Figures S7 to S23).

### Weather sub-model and mosquito habitat heterogeneity

The present model used a novel approach to model weather driven mosquito population dynamics that differs significantly from those used in previous studies [17, 23, 24, 33]. The weather sub-model uses temperature, rainfall and evaporation data from Cairns (available from the Australian Bureau of Meteorology http://www.bom.gov.au) as input and these weather data drive the mosquito population dynamics model component.

Temperature dependent parameters of the mosquito population dynamics model (Additional file 1: Figure S7) are determined similarly to the previous studies referenced above (described in detail in the Additional file 1 e.g., Table S1 and Figure S21). The effect of temperature on the mosquito population is assumed to be a global effect, which occurs to the same extent in all model cells. As in the previous studies, we assume that mosquito larvae develop in a density dependent manner, that is, their growth rate declines to a minimum as their density increases towards a maximum value (for further information see Additional file 1 pages S13 to S14 and Figures S12, S13, Table S2 and Equations S6 and S7).

To permit spatial heterogeneity within the mosquito population we assume that certain cells are better suited to support mosquito development than others. We define the minimum and maximum *capacity of a model cell to sustain mosquito larvae* (*L*_*min*_ and *L*_*max*_). The minimum capacity of a cell to sustain mosquito larvae is maintained throughout the year, irrespective of rainfall and accounts for artificially watered containers present in the cell and for those containers that are shielded from evaporation. Following rainfall (in the model this is the time when the amount of rain exceeds the amount of evaporation), a cell’s capacity to sustain mosquito larvae increases until it eventually reaches a maximum value (*L*_*max*_). We assume that *L*_*max*_ marks the point at which containers overflow and a cell is saturated with water, so that its capacity to sustain mosquito larvae does not increase further. We allow for a fraction of the immature mosquito stages to be washed out from overflowing containers (Additional file 1: Figure S11 and Equation S5). The capacity of a cell to sustain mosquito larvae thus fluctuates between the *L*_*min*_ and *L*_*max*_ values with rainfall and evaporation and cells can hold more or less mosquito larvae depending on rainfall, which in tropical Cairns varies between wet summers and dry winters. For detailed explanations of the weather sub-model refer to the Additional file 1 pages S9 to S15).

In contrast to previous dengue simulation models that use complicated container characteristics which need to be determined by field studies [15, 16, 18, 33], the approach adopted here is aimed at the use of geographic information system (GIS) data to estimate spatial distribution of mosquito habitats. While this may be less accurate than manual classification of each container in the modelled area, it has the benefit of allowing for transfer model to other geographic locations more readily.

It is assumed that two cell-specific geographical features are positively correlated with a *cell’s capacity to sustain mosquito larvae,* namely *i)* the degree of vegetation cover in a cell and *ii)* the number of dwellings per cell. This assumption is based on previous work [34, 35] and the positive association found between mosquito trapping data from Cairns and these two characteristics in the present study. We assume that the number of dwellings per cell is more important than vegetation, since it provides *i)* humans, who are the source for *Ae. aegypti* blood feeding and *ii)* human-made breeding habitats such as rainwater tanks, underground sumps, flower pots etc. [36]. Previous studies have shown significant correlations between vegetation cover and the abundance of *Ae. aegypti* breeding sites in urban settings [35], in the model the presence of vegetation cover adds an additional benefit to a cell’s capacity to sustain mosquito larvae, as it may prevent increased solar exposure and enable lower local rates of evaporation. Vegetation features may also provide additional breeding sites such as fallen palm tree fronds which may collect water.

We introduce a *breeding site abundance index (B)*, which is directly proportional to the *cell’s maximum capacity to sustain mosquito larvae (L*_*max*_ *~ B*) and use a relationship between *B*, the number of dwellings (*D*) and vegetation cover (*V*) in the form of Equation 1.

1 $$B=D+\mathrm{DV}+B_{\min}$$

A minimum breeding site index *B*_*min*_ is present independently from the coverage with dwellings and vegetation. Additional file 1: Figure S15 shows the distribution of the *breeding site abundance indices (B)* based on Equation 1 (Equation S8). *B* is normalized to between 0 and 1 as it is only a relative value that stands for the suitability of a cell for mosquito reproduction. We believe, that the general distribution of expected mosquito breeding sites is realistically represented by the resulting pattern (Additional file 1: Figure S15). For example, industrial areas have low breeding site indices (blue). Houses with significant amounts of surrounding vegetation, as in Parramatta Park (a suburb with a history of dengue outbreaks), have high breeding site indices (red).

As the process of assigning a mosquito breeding site abundance index to each cell does not rely on fitting-to-data, validation of this methodology was conducted using available trapping data from Cairns for the years 2006–2013, kindly provided by Scott Ritchie (James Cook University) and Peter Cook (Monash University). We relied only on the data collected using commercially available BG traps since these are considered to be reliable [37]. Note that the available trapping data were limited to a small proportion of the modelled area (see Additional file 1: Figure S18 for trap locations). For further information on the validation process using mosquito trapping data refer to the Additional file 1 (Pages S15-S18, Figures S15-S18).

### Calibration of the mosquito population dynamics model

**Figure 4:**
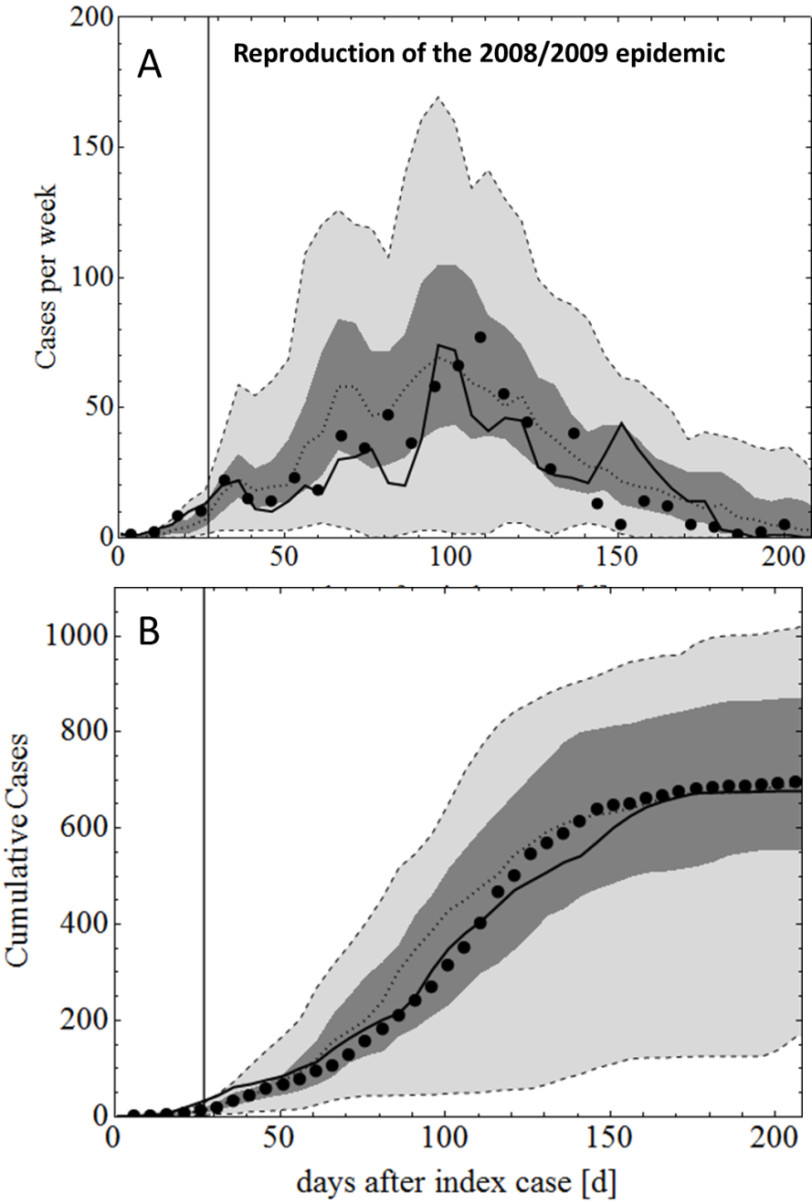
**Reproduction of the 2008/2009 epidemic.** Panel **A** pictures the weekly case numbers while panel **B** pictures cumulative case numbers. Black dots denote the observed weekly outbreak data, black solid lines denote the best stochastic realisation, dotted lines, dark grey and light grey areas denote the median, interquartile range and 95% confidence interval of 57 stochastic realisations, respectively. The black vertical lines indicate the onset of control interventions (day 27 after the index case). Some (3/60) stochastic realizations did not result in further transmission (index case being the only case) and these were excluded from the analysis. The median total number of *predicted* cases was 692 (172–1029), while the *observed* number of actual cases in the modelled area was 696.

The mosquito population dynamics model was calibrated using an observed mosquito density pattern as shown in Figure 4 (Additional file 1: Figure S20) using the weather data present in the same figure as an input.

The mosquito trapping data consisted of mean number of female *Ae. aegypti* captured by Biogents Sentinel traps (Figure 4; Additional file 1: Figure S20) from 09/10/2006 and 16/08/2008 [38]. During this time, no major dengue outbreaks were recorded in the modelled area, hence no significant mosquito control activities impacted on the mosquito population. A detailed description of the calibration process is given in the Additional file 1.

**Figure 5:**
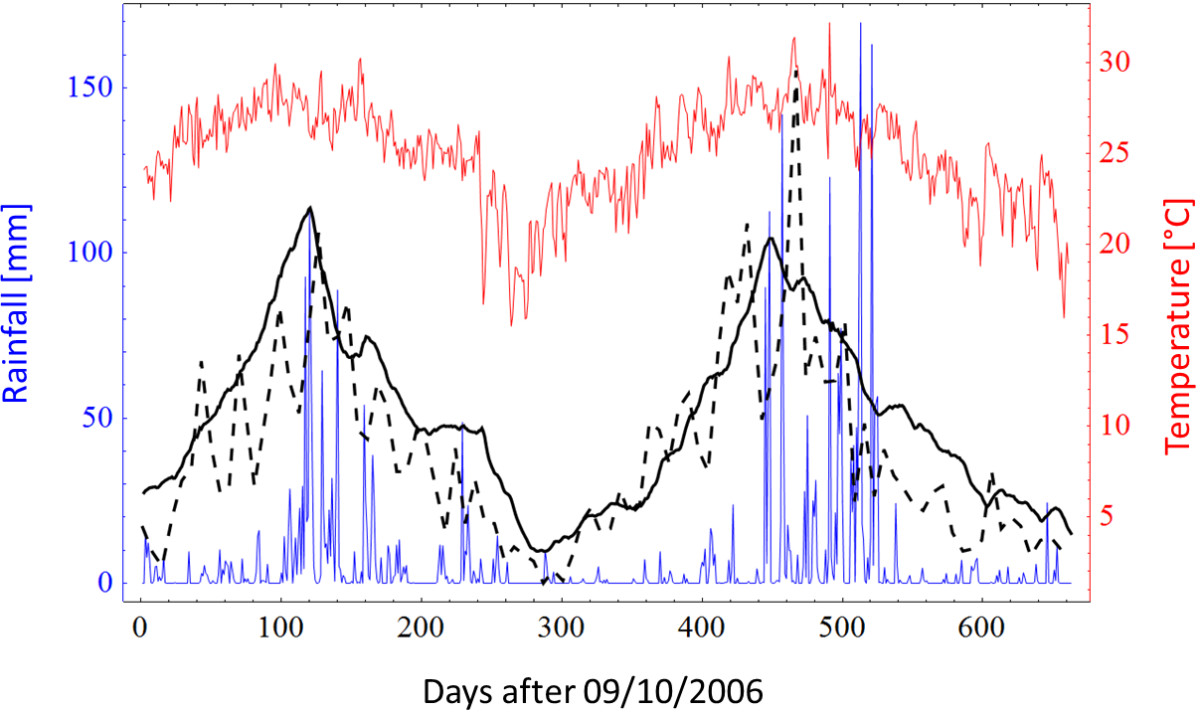
**Fitted Mosquito Density (solid black) versus observed density (dashed black).** Also shown are the input rainfall (blue) and temperature (red) data. Observed mosquito density was adapted from Azil et al. using data from BG traps [38]. Note that mosquito density is presented without a scale since it is the relative mosquito density which scales to an absolute mosquito density by adjusting L_max,_ as described in the Additional file 1.

Only the unknown or uncertain model parameters listed on page S22 of the Additional file 1 were used to calibrate the mosquito population dynamics model to the relative mosquito abundance across the entire modelling area as depicted in Figure 5 (Additional file 1: Figure S22). A random-walk Monte Carlo approach was used to identify the set of unknown or uncertain parameters that resulted in the best fit to the mosquito trapping data.

The mosquito trapping data provides a measure of *relative* mosquito abundance, therefore, in Figure 5 (Additional file 1: Figure S22) mosquito trapping data and the calibrated mosquito abundance curve are presented without a scale, since the absolute number of mosquitoes is unknown.

Variation of *L*_*max*_*,* which is the maximum cell capacity to sustain mosquito larvae, will not change the relative shape of the calibrated curve presented in Figure 5 (Additional file 1: Figure S22). However *L*_*max*_ can be used to scale the absolute mosquito population based on the assumption that all modelled cells should follow a similar mosquito density pattern, though some are more suitable to sustain mosquito reproduction than others. Furthermore, it is assumed that absolute mosquito density should reflect an average female mosquito number of 8–24 *Ae.aegypti* per property, as estimated from mosquito trapping and computer simulation studies in Cairns [39, 40]. Since the modelled area contains approximately 28,000 properties, *L*_*max*_ was chosen to obtain an average mosquito number of around 2.24 × 10^5^ to 6.72 × 10^5^ mosquitoes in the modelled area (about 8–24 mosquitoes per dwelling). A resulting graph showing absolute mosquito abundance is shown in Additional file 1: Figure S23.

The calibrated version of the mosquito population sub-model allows for the calculation of temperature and rainfall dependent mosquito profiles for any year for which weather data are available. In years where there are large dengue outbreaks, the associated vector control programme will impact on the mosquito population beyond what may be calibrated with the pure mosquito population dynamics model presented here. Hence, only the coupled mosquito population dynamics, dengue transmission and dengue control model can be used to estimate mosquito population curves in years with significant dengue outbreaks.

### Dengue transmission

We use a standard SEIR transmission model as done in other studies, including those modelling dengue transmission [16, 25–29]. There are two *components* to the transmission model *i)* the human one and *ii)* the mosquito one. The dengue transmission sub-model is described in detail in the Additional file 1 (pages S24-S26).

At any given time individual humans will be in one of four states, namely *S* susceptible, *E* exposed/infected, *I* infectious and *R* recovered/immune. The model allows for the possibility that individuals become infectious but the disease is not diagnosed or progresses asymptomatically (we do not further discriminate between the causes of non-detection; it may be due to misdiagnosis or the absence of symptoms). This results in individuals being in one of two infectious states, *I*_*S*_ symptomatic or *I*_*A*_ asymptomatic. A schematic representation of the human population model is presented in Additional file 1: Figure S24 (and simplified in Figure 3).

Both A1 and A2 stage mosquitoes (Additional file 1: Figure S7) bite and transmit dengue virus. The infection process in the mosquitoes does not include a recovered/immune state as it is assumed that mosquitoes remain infected until they die. A schematic representation of virus progression in the mosquito is shown in Additional file 1: Figure S25 (and simplified in Figure 3).

Note that none of the transitions between human or mosquito states described above are simple first order kinetics. Probabilities of infection from humans to mosquitoes *P*_*H→M*_ and from mosquitoes to humans *P*_*M→H*_ will depend on the number of humans and mosquitoes co-located in a cell, resulting in these transitions being stochastic processes based on sampling from binomial distributions. Intrinsic incubation times and human recovery rates are *gamma* distributed which makes early progression less likely [41]. The extrinsic incubation periods in mosquitoes (Additional file 1: Figure S26) are sampled from temperature dependent *log-normal* distributions based on studies by Chan et al. [42].

### Control measures

**Figure 6:**
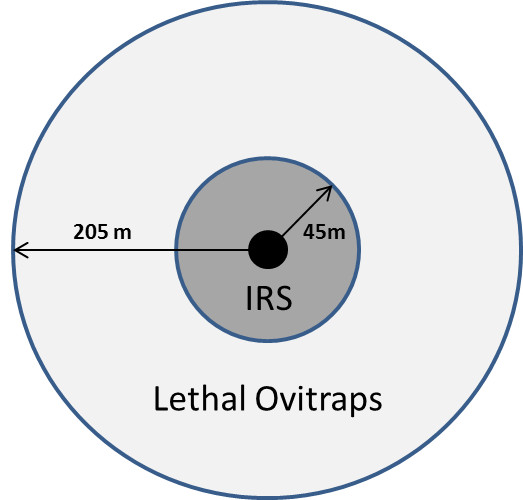
**For any reported/symptomatic dengue case, two control mechanisms are invoked.** In-house residual spraying and larvicide treatment are used within the case cell (black dot) and adjacent cells (dark grey). Lethal ovitraps are placed in the wider radius around the case cell.

The model implements conventional means of vector control that are used in Cairns to eliminate dengue spread during outbreaks. These include *i)* within-house residual spraying (IRS) and larvicide treatment around case properties and the properties immediately adjacent and *ii)* the use of lethal ovitraps to kill egg-laying adults in extended areas around case properties. The control model is represented schematically in Figure 6 (Additional file 1: Figure S27).

It is assumed, that, following report of a case (i.e. that an infected, symptomatic case has occurred in a cell) IRS and ovitrap distribution would commence with an average lag time of 7 days [10].

IRS and larvicide treatment in the immediate vicinity of a case is known to be highly effective, killing an estimated 90% of all adult and immature mosquito stages present in the case cell and the 8 surrounding cells per day [43]. In the model it is assumed that the effect remains active for 6 weeks and affects all mosquitoes entering the cell during that time.

Lethal ovitraps have also been shown to be effective in controlling *Ae. aegypti.*[44]. Their deployment is faster than the more labour intensive IRS and larvicide treatment [45]. It is assumed that ovitraps are deployed at a certain rate per day in the cells within a 200 m radius of a case (depending on the amount of resources committed to fight a dengue outbreak, this rate may be high or low). Ovitraps kill approximately 20-30% of adult mosquitoes per cell per day [44]. The overall effect of lethal ovitraps is reduced due to the emergence of new mosquitoes, as we assume that lethal ovitraps only target the adult stage.

It is assumed that there are limitations on coverage and number of IRS premises treated and ovitraps deployed each day, depending on the size and number of available dengue response teams (i.e. human resources). If the required number of cells to be treated exceeds the maximum number treatable per day, the remaining cells that are left untreated are carried over to the next day.

The parameters were chosen to be consistent with the current dengue response plan of Queensland Health [45]. However, one of the capabilities of the model is to allow an analysis of alternative control strategies. Table 1 outlines all parameters used in the control part of the model.

**Table 1:** **Parameters used in the vector control part of the model**

***IRS + Larviciding***		
Effect radius	45 m	QH Dengue Management Plan
Efficacy	90% per day	QH Dengue Management Plan, [43]
Duration	6 weeks	[12]
Maximum cells treatable per day	15	[37]
***Lethal Ovitraps***		
Effect radius	205 m	QH Dengue Management Plan
Efficacy	20% per day	[44]
Duration	4 weeks	[44]
Maximum cells treatable per day	150	[37]

**Figure 7:**
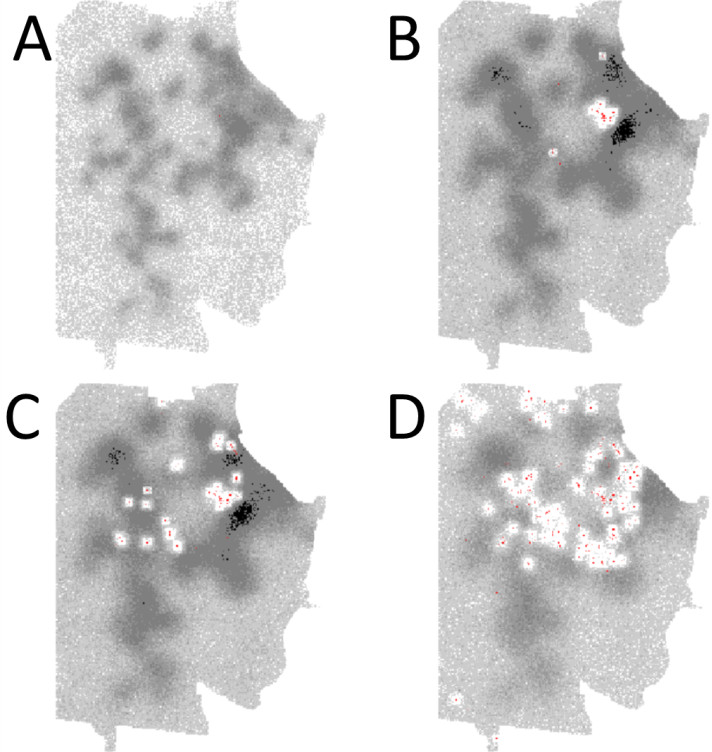
**Example of the effect of control on mosquito density during a simulated 2003-like dengue outbreak.** The sequence of images (A-D) shows a simulated outbreak (based on the 2003 outbreak) on day 1 (Panel **A**), day 60 (Panel **B**), day 120 (Panel **C**) and day 180 (Panel **D**) The red markers indicated dengue case locations. The grey layer indicates local mosquito density in each model cell (white = 0 mosquitoes, light grey = 1–10 mosquitoes, dark grey = 10–50 mosquitoes, black = 50+ mosquitoes per cell). Since the outbreak occurred during a time when the seasonal mosquito density was still rising, the background mosquito density is lower in Panel A than in the other panels. A video of the entire sequence is supplied as Additional file 2. It can be seen how vector control significantly affects the mosquito numbers over the course of the outbreak.

An example of the effect of vector control on the mosquito population in the model is illustrated in Figure 7 (Additional file 1: Figure S28).

Additional file 2: Animation of simulated 2003 Dengue outbreak, showing mosquito density in grey and human Dengue cases in red.(MP4 700 KB)

Further information on the dengue control sub-model may be found in the Additional file 1.

### Model calibration using dengue outbreak data

**Figure 8:**
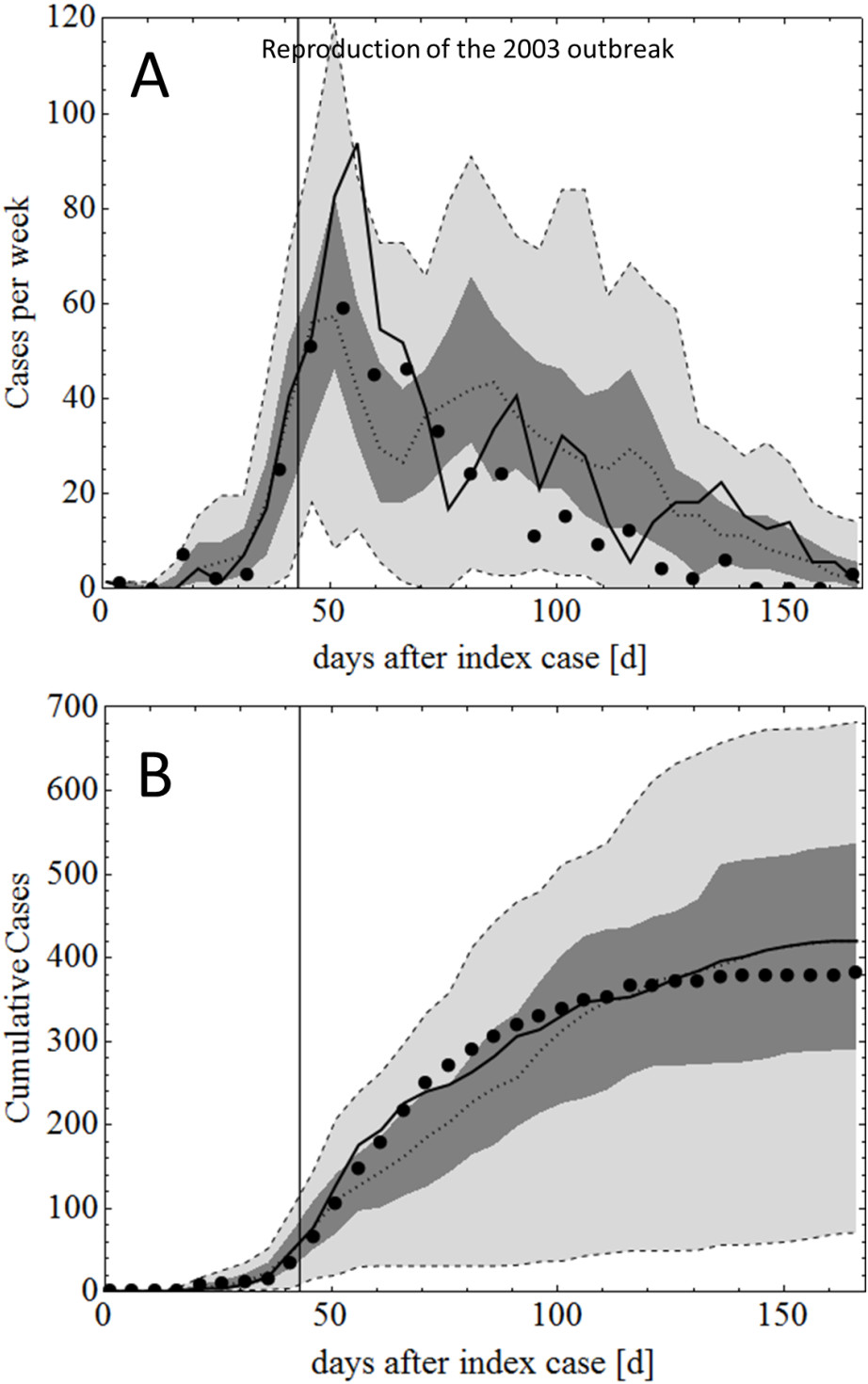
**Model Calibration using data collected during the 2003 DENV2 outbreak.** Panel **A** pictures the weekly case numbers. Panel **B** represents the cumulative number of cases**.** Some (7/60) stochastic realizations did not result in further transmission (index case being the only case). These were excluded in the present analysis. Black circles denote the observed outbreak data; black solid lines denote the best stochastic realisation; dotted lines, dark grey and light grey areas denote the median, interquartile range and 95% confidence interval of the remaining 53/60 stochastic realisations, respectively. The black vertical lines show the onset of control interventions (day 43 after the index case). The median total number of *predicted* cases was 420 (17–804), while the *observed* number of cases was 386.

The dengue transmission and control components of the model were calibrated by fitting to a well-documented DENV 2 dengue virus outbreak in Cairns in 2003 [10]. This calibration was performed by running repeated simulations and gradually adjusting parameters until a close match between simulated and actual outbreak data was achieved. This process occurred as follows: firstly, uncertain dengue specific parameters (e.g. human to mosquito and mosquito to human transmission probabilities) were used to calibrate the model to the *unmitigated* initial phase of the outbreak, where dengue spread was not affected by subsequent control. Secondly, the model was calibrated using the entire 2003 outbreak data by adjusting other uncertain parameters related to the control measures, such as the lag between infection of a case and control measures targeting the location of that case. Figure 8 shows the resulting calibrated outbreak curves.

### Sensitivity analyses

Sensitivity analyses for the main model parameters are presented in Table 2. It is shown that, as can be expected, the model is highly sensitive to the mosquito biting rate. It can also be seen from the sensitivity analyses that both human and mosquito movement are important factors that drive the spread of infection.

**Table 2:** **Sensitivity of the model to A: Main model parameters and B: Alternate settings**

A				
	2003		2008/2009	
**Parameter variation**	+50%	-50%	+50%	-50%
Biting rate	**1261** (717–1719)	**48** (10–117)	**2471** (1984–2930)	**14** (2–61)
Asymptomatic fraction	**362** (172–527)	**516** (165–818)	**743** (370–938)	**698** (100–1086)
Mosquito to human transmission probability	**461** (138–746)	**217** (18–349)	**794** (4–1183)	**194** (3–390)
Human to mosquito transmission probability	**564** (291–828)	**355** (160–538)	**880** (501–1355)	**439** (25–779)
**B**				
**Alternate Setting**				
No mosquito mobility	**34** (6–152)		**99** (3–421)	
No human mobility	**104** (28–184)		**19** (6–58)	
No state of emergency	N/A		**1479** (440–2202)	
Index cell with low mosquito density	**12** (1–26)		**4** (1–30)	

**Figure 9:**
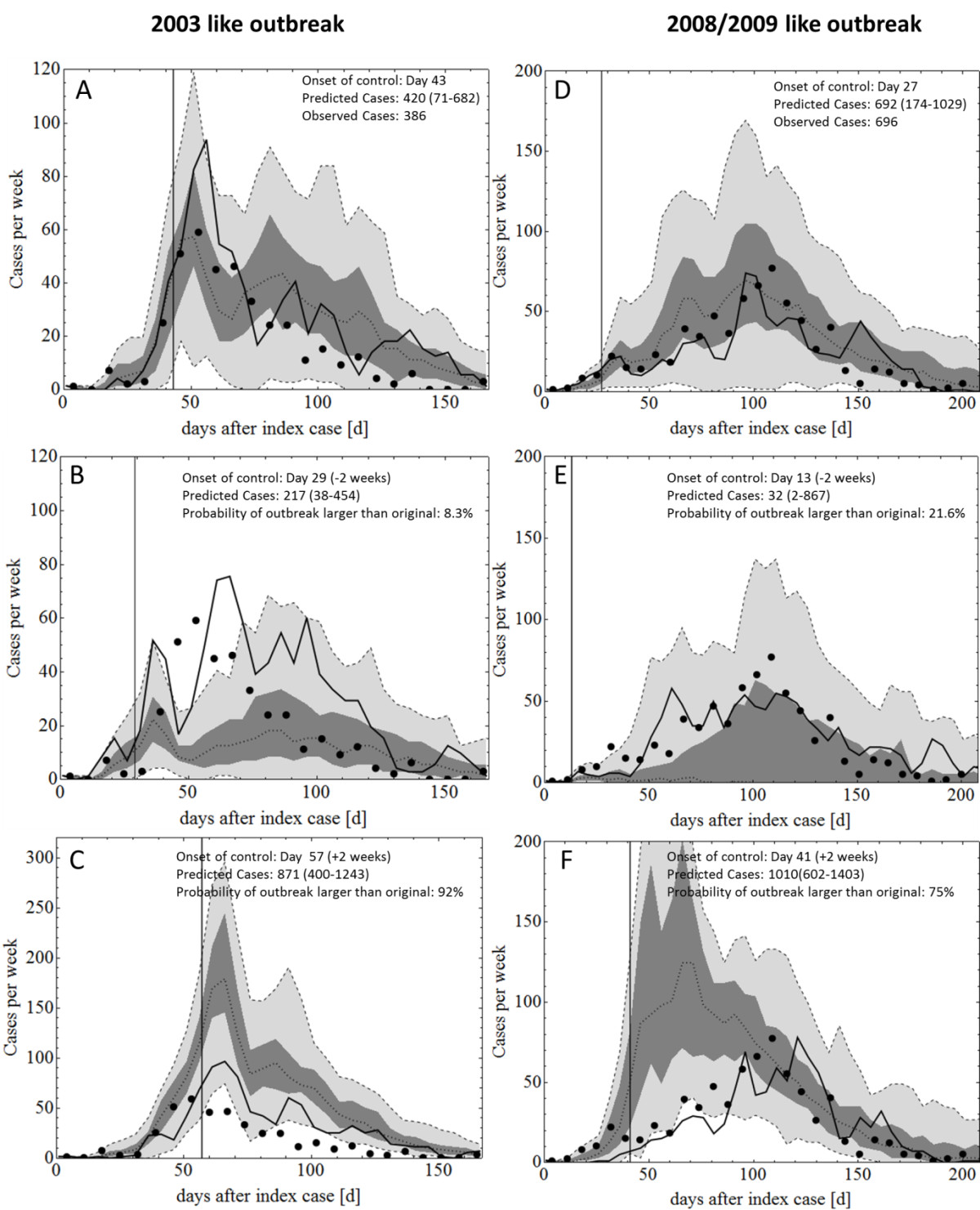
**Variation of the initiation time of vector control measures.** Panels **A** and **D** show simulations that reproduce the original 2003 and 2008/2009 outbreaks. Panels **B**,**C**,**E** and **F** show simulated 2003 and 2008/2009 like outbreaks where the onset of control was varied by +/-2 weeks from the original start day. In both cases, variation of the control start day had a significant effect on the outbreak curve and final case numbers, confirming previous studies, such as [46]. In the plots, black dots are observed outbreak data, solid black lines are best stochastic realisations, dotted lines are the medians of 60 simulations, dark gray shaded areas are interquartile ranges, light gray shaded areas are 95% confidence ranges. Note that the predicted cases shown in Panels **A**-**F** are the median case numbers of 60 stochastic realizations. Often there is a considerable spread, e.g. in Panel **E** the median (middle dotted line, which lies almost on the x-axis) predicted number of cases is 32 but in rare cases, case numbers can exceed 800.

The values in the table appearing in bold are medians of 60 simulations, with 95% confidence intervals given in parentheses. For comparison the predicted case number without any parameter variation for 2003 and 2008/2009 were 420 (71 – 682) and 692 (174 – 1029) respectively (see e.g. Figure 9 A and D in the main manuscript).

## Results

### Model validation

Following calibration of the model using the 2003 dengue outbreak data, the model was applied to a subsequent, larger epidemic which occurred in Cairns in 2008/2009 (see Figure 4). The 2008/2009 epidemic caused nearly 700 cases in the modelled area, twice as many as the 2003 outbreak. Ritchie et al. [7] discuss several factors that are thought to have contributed to the explosive expansion of the epidemic in late 2008 and its rapid collapse in April 2009. These contributing factors are: *i)* climatic factors (an unusually warm period in November 2008), *ii)* dengue virus related factors, specifically the shorter extrinsic incubation period of the DENV3 strain that caused the 2008/09 epidemic, compared to the 2003 DENV2 outbreak [7], *iii)* human factors, specifically the increased human movement over the Christmas period and *iv)* the declaration of the outbreak to be an epidemic, invoking a significant expansion of control measures in January 2009 [7].

Using the simulation model it was demonstrated that all of these factors may have acted together to cause the larger scale of outbreak which occurred in 2008/2009 compared to previous dengue outbreaks in Cairns, though some of these factors may have played a more significant role than others. Applying the 2008/2009 temperature profile resulted in an average predicted extrinsic incubation period that was approximately 1 day (0.75 days) shorter than that in 2003. Furthermore, the rainfall pattern in 2008/2009 did not result in a greater predicted mosquito population than in 2003, which is in agreement with mosquito trapping data [7]. As a result, climatic factors alone did not increase the simulated case numbers significantly.

In contrast, simulation experiments demonstrated that the shorter extrinsic incubation period of the specific DENV3 strain that caused the 2008/2009 epidemic (as discussed in [7]) allowed a transmission cycle to be completed in approximately 10 days compared to 17 days in 2003, causing a considerable rise in case numbers and an epidemic that could not be controlled using the initial (2003-based) control measures. The modelled outbreak with significantly increased control measures activated indicated that it was the expansion of control interventions starting in January 2009, following declaration of the outbreak as an epidemic, which caused the decline in weekly case numbers and eventually a rapid collapse of the outbreak. Experiments using the model with the control measures set at the lower 2003-like level resulted in a much longer outbreak which relied on seasonal weather changes before the outbreak was contained, confirming the need for the increased response measures. Increased human movement over the Christmas period (in the model, the month of December) only slightly altered the overall case numbers (by an average of about 50 cases in total).

Table 3 shows the conditions in 2008/2009 that resulted in the best reproduction of the epidemic using the simulation model. The shorter EIP had by far the most significant effect Simulated weekly cases and cumulative cases for the 2008/2009 epidemic are shown in Figure 4.

**Table 3:** **Differences between the 2003 and 2008/09 outbreak scenarios accounted for in the present study**

	2003	2008
Index case cell location	Paramatta Park	Cairns North
Onset of Control	day 43	day 27
Mosquito population	based on 2003 weather	based on 2008 weather
Extrinsic incubation period	4-15 days	2-6 days
Human movement	3-4 times per week	6-7 times per week for Dec. 2008
Interventions	constant after day 43	increased after day 60 (epidemic)

Successful application of the model in reproducing the 2008/2009 epidemic can be seen as a validation of the overall model as well as the settings used for the key model parameters, which were uncertain prior to calibration with the earlier 2003 outbreak data. Time lapse representations of the 2003 and 2008/2009-like outbreaks are presented in the Additional file 2 and Additional file 3, respectively.Additional file 3: Animation of simulated 2008/9 Dengue outbreak, showing mosquito density in grey and human Dengue cases in red.(MP4 909 KB)

Following its development and testing, the model was used to investigate how changes to control measures affect 2003-like and 2008/2009-like outbreaks, providing guidance for the management of future outbreaks.

### Effect of variation of activation of control measures

A study by Vasquez-Prokopec et al. [46] investigated the consequences of delayed onset of vector control measures for the same 2003 and 2008/2009 outbreaks, using a simpler non-spatial model and found a dramatic increase in total case numbers as a result of delays in the initiation of control measures [46]. Similar scenarios were investigated using the simulation model presented here, where two alternative control scenarios were compared with the original 2003 and the 2008/2009 outbreaks. These scenarios were characterised by either a 2 week *earlier onset* of (otherwise unchanged) control measures or a 2 week *delayed onset* of the control measures. Figure 9 shows the predicted scale of the outbreak when initiation of control measures is varied +/-2 weeks from the actual starting time.

The results are in agreement with the previous study by Vasquez-Prokopec et al. [46], showing that variation of the start date of vector control had a major impact of the overall scale of the simulated outbreaks. In simulations of 2003-like outbreaks, initiating vector control 2 weeks earlier than that which occurred in the actual outbreak reduced the median number of cases by ~50%. Starting control 2 weeks later than the original start date approximately doubled the predicted median number of cases. In all scenarios the control measures were able to reduce weekly case numbers by a similar amount and within a similar time-frame as that observed in the original outbreaks (approximately 150 and 200 days respectively for 2003 and 2008/2009).

In the simulations of a 2008/2009-like epidemic, a 2 week earlier onset of vector control reduced the median case number to 32 (from 692), a reduction of nearly 20 fold. This large difference is due to the very early onset of control (day 13 after index case was diagnosed), resulting in onward transmission having failed to spread beyond a 200 m radius of the index case. The location of the index case was an area where control measures were actively applied, eradicating almost all mosquitoes within a couple of days (compare e.g., Figure 7 or Additional file 2 and Additional file 3). As with the 2003 simulations, lengthening the unmitigated phase of the 2008/2009-like epidemic approximately doubled case numbers but did not prolong the overall duration of the outbreak, which corresponds with previously published estimates by Vasquez-Prokopec et al. [46].

### Effect of increased interventions

**Figure 10:**
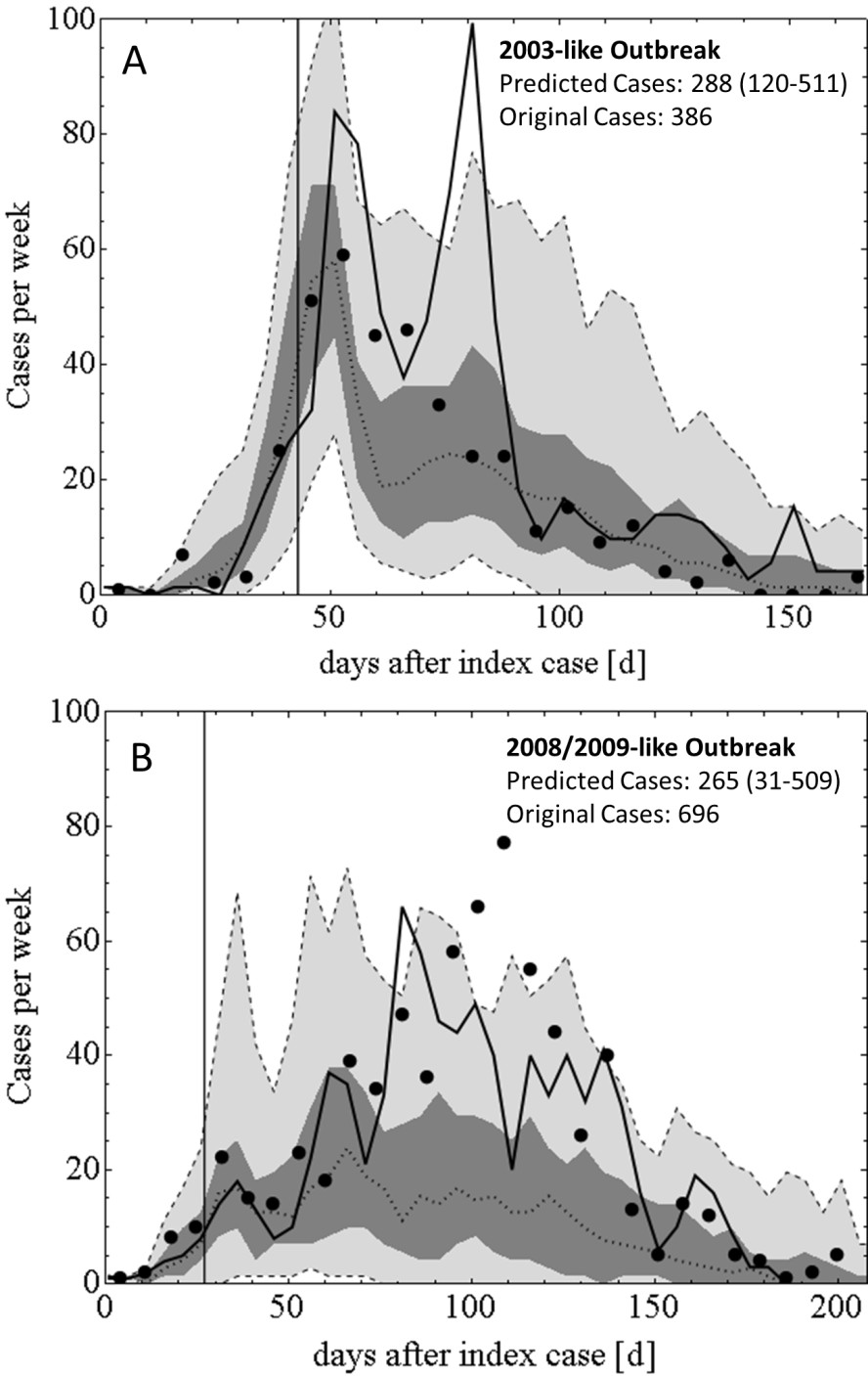
**Effect of expanded vector control on the scale of a 2003-like outbreak (Panel A) and a 2008/2009-like outbreak (Panel B).** Black dots are the observed data, solid black lines are the best stochastic realisations, black dotted lines the median case numbers, dark grey areas are interquartile ranges, light grey areas are 95% confidence intervals.

The model was further applied to investigate the effect which expanded IRS spraying and ovitrap placement would have on 2003-like and a 2008-like outbreaks, where the date of intervention initiation remains unchanged. These experiments were performed as an illustration of the capability of the model to replicate the effect of altered control strategies. The radius of IRS application around the case property was extended to 100 m and the radius of lethal ovitrap placement was extended to 300 m. Furthermore, the capacity for IRS and lethal ovitrap placement was increased from 15 per day to 30 per day and from 150 per day to 300 per day, respectively. The results of the experiments are presented in Figure 10.Figure 10 illustrates that expanded control measures substantially reduce the scale of the simulated outbreaks, but that they do not reduce overall case numbers as effectively as shortening the delay in the onset of control, as described above. Such increased control measures caused a ~30% reduction in predicted case numbers in the 2003-like scenario and a ~60% reduction in the 2008/2009-like scenario.

#### Sensitivity analysis

Sensitivity analyses of the model parameters such as biting rate, fraction of asymptomatic infections, mosquito to human transmission probability and human to mosquito transmission probability were conducted. The values of the relevant model parameters have been adjusted, and the simulation model rerun, to determine the sensitivity of the model to particular parameter settings. While particular parameters were adjusted, no model recalibration took place. The outcomes, for the 2003 and 2008/2009 outbreaks, are presented in Table 2.

## Discussion

The model has been customised for the Cairns setting, however we believe that the underlying modelling methods will be applicable to other settings. In the future, an aim will be to explore how the modelling approach presented here can be applied to other urban dengue transmission settings such as areas with endemic dengue, areas with different population structures, and to larger cities. With appropriate re-parameterisation, the model may also be applicable to other *Ae. aegypti* (and *Ae. albopictus*) vectored viruses such as chikungunya [47]. The transfer of the model structure to other geographic locations will depend upon on the availability of data, which was extensive in the case of Cairns (a table of required data sources is given in Additional file 1: Table S4). Furthermore, a transfer of the model to a location endemic for dengue may also require changes to the model structure by introducing measures for herd immunity or properties of multiple circulating dengue strains [34].

This study highlights a direction of vector-borne infectious disease modelling away from compartmentalised population-based, spatially-homogenous approaches towards individual-based, spatially-explicit techniques, a move also evident in modelling studies focused on other vector-borne diseases such as malaria [48, 49].

A key goal in developing the model has been to capture the most important features of mosquito population dynamics, relevant human behaviour, dengue transmission and control without making the model overly and unnecessarily complex. It has been recognized that a challenge with developing complex models such as that presented here is to avoid the temptation to make them unnecessarily overcomplicated, as discussed by Basu et al. [50]. The model presented here simplifies certain aspects of the physical world being modelled, usually due to limitations of data availability. These limitations include: *i)* lack of detailed mosquito trapping data prevented the development of a more detailed mosquito density layer, *ii)* simplified human movement (e.g., the use of semi-random movement is only a rough approximation of reality), *iii*) simplified mosquito movement (it may be that mosquitoes do not fly randomly but are directed by various factors such as availability of humans, wind direction etc.), *iv)* simplified effect and dynamics of vector control (lag time between a case and local control may vary on a case-by-case basis, properties may only be partially treated etc.), *v)* abstraction of mosquito breeding sites (we do not model every individual container that may serve as a breeding site as with the CIMSIM/DENSIM models) [38]. The model is less detailed in the way it accounts for the presence of mosquito breeding sites compared to the CIMSIM/DENSIM approach, yet in the light of recent findings that some of the detail incorporated into the CIMSIM mosquito population dynamics model may be superfluous this approach seems justified [18]. The validation of the model using high-quality data from a different outbreak to that used to calibrate uncertain model parameters suggests that the level of abstraction adopted is appropriate, and results in a modelling framework which will be capable of use to examine the effectiveness of new intervention strategies as well as helping to explain phenomena contributing to the conditions necessary for dengue to become endemic in Cairns-like settings.

This study describes the development of a complex, spatially-explicit and individual-based dengue transmission model, and makes use of spatio-temporal dengue outbreak data in a novel way to demonstrate that such a model is capable of simulating dengue outbreaks of the type that occur in Cairns, along with the intervention measures used to control them. The study clearly shows the need for high quality outbreak field data to inform and parameterize the model. If such data are available, as was the case in the present study, such a simulation model can be adequately calibrated.

This study presents a dengue modelling framework and highlights how key features of the physical system may be represented in a simulation model. While this modelling methodology has been validated by showing that the system as a whole reproduces the outbreak dynamics of a single dengue epidemic, the availability of new field data will permit further model validation and future model refinement, aiming at making the system more physically realistic. New field data collection will permit further validation of the system as a whole, and for the separate validation of each sub-model that makes up the overall modelling environment.

Limitations of this study include the fact that the model assumes a population centre that experiences *episodic* dengue outbreaks, and not populations where dengue is endemic. As such, it is assumed that the human population has no immunity to dengue, and that only one dengue strain is present during the outbreak. However the methods used to incorporate the necessary sub-models within the Cairns dengue model will also be applicable to the construction of models where dengue is endemic. Another limitation of the study was that *Ae. aegypti* trapping data was only available for a limited part of the Cairns urban area; availability of comprehensive mosquito trapping data would allow for further refinement of the spatially-heterogeneous vector habitat sub-models. Based on the *Ae. aegypti* trapping data, it was assumed rainfall was the main driver of the availability of larval habitats, and thus adult population densities. In other geographical locations (for example, where rainfall is uniform throughout the year) other factors may determine the availability of larval habitats and *Ae. aegypti* population dynamics. While detailed data on human movement is sparse and our use of a daily cycle of moving to work or school captures regular patterns of human movement within a community, our simple semi-random model of other types of (longer range) human movement could be refined if new reliable experimental data becomes available. Finally, we note that each sub-model which contributes to the overall model is based on the best available data and on expert-informed assumptions where experimental data is not available. Further analyses could be conducted to explore the sensitivity of the model to alternative plausible assumptions about, for example, human movement patterns, or factors determining the spatial heterogeneity of larval habitats.

## Conclusion

This study was aimed at developing an individual-based, spatially-explicit model for dengue transmission in an urban environment. This research was facilitated by prior development of mosquito population dynamics models by others [15–25, 38, 51, 52] and research studies describing and quantifying *Ae. aegypti* entomology and dengue outbreaks, virus properties and the impact of vector control in Cairns [7, 10, 12, 13, 36, 39, 40, 44, 52–55]. Specific goals of the study were to produce a simulation modelling framework that could (a) make use of rich dengue outbreak data sets for calibration and validation; (b) would be capable of investigating hypotheses explaining the size of the 2008/2009 Cairns outbreak; and (c) would incorporate phenomena essential to the prediction of future dengue outbreaks and analysis of dengue control measures, viz. weather-dependent vector population dynamics, spatially heterogeneous vector habitat, spatially targeted vector control, and human host movement.

In order to achieve these goals, the resulting model contains a number of key features. These include: *i)* human and mosquito movement and realistic population structures, *ii)* weather-dependent (temperature and rainfall) spatially heterogeneous mosquito population dynamics, *iii)* geographically and demographically- dependent mosquito abundance, *iv)* spatially- explicit vector control, *v)* model calibration using outbreak data and *vi)* model validation against further outbreak data. The resulting simulation model is complex, consisting of several interacting sub-models. However, this complexity is *necessary*, and is required in order to represent the inherently complex physical phenomena which contribute to dengue transmission and the scale and timing of dengue epidemics.

The simulation results replicating the 2008/2009 Cairns epidemic presented in this study support several hypotheses (formulated previously) aimed at explaining the large-scale epidemic which occurred in 2008/2009 [7]. Specifically, while warmer weather and increased human movement had only a small effect on the spread of the virus, a shorter virus strain-specific extrinsic incubation time can explain the observed explosive outbreak of 2008/2009 [7]. In agreement with previous studies, the simulation results presented here highlight the importance of rapid diagnosis of potential index cases and prompt initiation of vector control, as presented above in Figure 9[46].

This study, in combination with several previous dengue modelling studies, highlights the importance of spatial and individual-based modelling approaches in order to account for local differences in mosquito and human density and differences in human movement behaviour depending on age and other factors [18, 31, 56]. This study confirms some previously known spatial phenomena, such as human movement and mosquito movement, which facilitate dengue spread (see sensitivity analyses in Table 2, where such movement has been excluded, dramatically reducing simulated case numbers) [31, 56].

The availability of this model will allow further investigation of the effect of different intervention strategies which target various stages of the transmission cycle, such as the effect of potential dengue vaccines, and may also assist with predicting the effect of the release of *Wolbachia* infected mosquitoes on the native mosquito population, as is currently occurring in Cairns [57].

## Electronic supplementary material

Additional file 1: Detailed description of all model components.(DOC 3 MB)

Below are the links to the authors’ original submitted files for images.

Authors’ original file for figure 1

Authors’ original file for figure 2

Authors’ original file for figure 3

Authors’ original file for figure 4

Authors’ original file for figure 5

Authors’ original file for figure 6

Authors’ original file for figure 7

Authors’ original file for figure 8

Authors’ original file for figure 9

Authors’ original file for figure 10
