# Supplementary material for: A spatial simulation model for dengue virus infection in urban areas
Source: BMC Infect Dis. 2014 Aug 20;14:447. doi: 10.1186/1471-2334-14-447 (PMC4152583; doi:10.1186/1471-2334-14-447)
Supplement: Supplementary file 3 — Additional file 1: Detailed description of all model components.(DOC 3 MB) [file 12879_2014_3752_MOESM1_ESM.doc]

**A Spatial Simulation Model for Dengue Virus Infection in Urban Areas – Additional File 1**

Complete Model Description

Contents

[Human Population Model S3](#__RefHeading___Toc377569002)

[Spatial Resolution S3](#__RefHeading___Toc377569003)

[Temporal Resolution S3](#__RefHeading___Toc377569004)

[Individual Human Attributes S3](#__RefHeading___Toc377569005)

[Human age distribution and implications for human movement S3](#__RefHeading___Toc377569006)

[Distribution of human hosts in the modelled area S4](#__RefHeading___Toc377569007)

[Allocation of individuals to workplaces and schools S7](#__RefHeading___Toc377569008)

[Human Movement S7](#__RefHeading___Toc377569009)

[Mosquito Population Dynamics Model S9](#__RefHeading___Toc377569010)

[Overall Model Structure S9](#__RefHeading___Toc377569011)

[Temperature dependent independent parameters S10](#__RefHeading___Toc377569012)

[Adult mosquito death S11](#__RefHeading___Toc377569013)

[Rainfall and evaporation dependent parameters S11](#__RefHeading___Toc377569014)

[Population density dependent larval parameters S14](#__RefHeading___Toc377569015)

[Sensitivity analysis in the Hill parameter space S16](#__RefHeading___Toc377569016)

[Spatial Heterogeneity of Breeding Sites S16](#__RefHeading___Toc377569017)

[Adult mosquito flight S20](#__RefHeading___Toc377569018)

[Fitting the Mosquito Population Dynamics Model S21](#__RefHeading___Toc377569019)

[Virus transmission model S25](#__RefHeading___Toc377569020)

[Infection and virus incubation for humans S25](#__RefHeading___Toc377569021)

[Infection and virus incubation for mosquitoes S26](#__RefHeading___Toc377569022)

[Dengue Control Model S28](#__RefHeading___Toc377569023)

[Tools for vector control S28](#__RefHeading___Toc377569024)

[Resource constraints S28](#__RefHeading___Toc377569025)

[List of necessary datasets S31](#__RefHeading___Toc377569026)

[References S32](#__RefHeading___Toc377569027)

**Supplementary Tables**

Table S1 ……………………………………………………………………………S22

Table S2 ……………………………………………………………………………S22

Table S3 ……………………………………………………………………………S29

Table S4 ……………………………………………………………………………S31

## Human Population Model

The model of the human population developed in this study is based on previous work by Milne et al., who developed individual based model for pandemic influenza transmission in an urban setting.

### **Spatial Resolution**

The modelled area is divided into square cells with a 30 m x 30 m area. This uniform grid was chosen to enable the convenient implementation of adult mosquito flight, which is mostly limited to within 200 m of a mosquito’s place of emergence. The 30 m x 30 m spacing also represents the dimensions of a standard housing block in Cairns. This spatial resolution is also used to distribute human individuals across the modelled area by allocating households to the respective spatial cells. The exact process of how human hosts were distributed to individual cells is described later in this document.

### **Temporal Resolution**

We divide each day into four distinct time intervals, each of 6 hour duration. Individuals may move between different cells on some of these time steps (similarly, mosquitoes may fly to neighbouring cells during certain time intervals). Mosquito flight activity is based upon studies that indicate *Ae. aegypti* flight is concentrated to early morning and late afternoon. The time intervals are:

1. **Morning (3 am - 9 am):** all humans are in their home cells (and mosquitoes fly)
2. **Daytime (9 am - 3 pm):** depending on their age, humans are either in their home cells or the cells associated with work and school attendance (no mosquito movement)
3. **Evening (3 pm - 9 pm):** humans are in their home cells or visiting other residential, commercial or parkland cells (and mosquitoes fly)
4. **Night (9 pm - 3am):** all humans are in their home cells (no mosquito movement)

### **Individual Human Attributes**

Each individual in the model has a number of attributes:

1. **Age:** Age determines an individual’s movement behaviour as described below.
2. **Home Cell:** A home cell is the cell on the model grid in which the individual’s residential property is located. Since the modelled area is made up of 900 sqm square cells as the core spatial units, a home cell can contain 1 or more residential properties, but will rarely contain more than 20 humans.
3. **Work Cell or School Cell:** A work or school cell is the cell on the model grid in which an individual’s workplace or school is located.
4. **Infection Status:** Each individual can assume one of the four states of the classic SEIR model for infectious diseases : S-susceptible, E-infected, I-infectious and R-recovered. Since dengue results in life-long strain specific immunity, recovered is equivalent to ‘removed from the transmission process’ in the present model.

### **Human age distribution and implications for human movement**

One of the core assumptions of the present human model is that the population is constant, i.e., there are no births and deaths in the human population. We believe that this is a reasonable assumption since the time span being modelled is only of the order of 6 months. Each individual is assigned a specific age, in accordance to the age distribution in the model area as accessed through the Australian Bureau of Statistics for the 2011 census in Cairns (www.abs.gov.au). The age distribution of all individuals in the present model is shown in Figure S1.


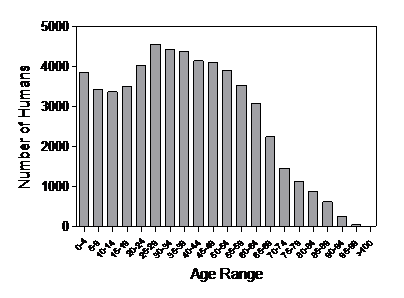


**Figure S1: Overall age distribution of the modelled human population**.

We make no assumptions about different behaviours of males versus females (e.g., different rates of unemployment) and thus do not separate by gender.

Previous studies have shown that human movement is an important factor facilitating the spread of vector borne infectious diseases including dengue. . In the model the age of an individual is directly related to the type of movement the individual can perform. We further simplify the age distribution to ranges that directly translate into different human movement behaviours. This classification can be summarized as:

1. **0 - 4 years:** infants/toddlers mostly stay at home except occasional random visits of properties other than the home property
2. **5 - 19 years:** school age children and adolescents attend their respective schools; we further separate this cohort in to 5-9 years for primary school and 10-19 years secondary school. Children attending schools are assigned to their nearest primary or secondary school and move in between their home location and the school on every weekday
3. **20 - 64 years:** working age adults go to work at commercial or industrial properties. We account for an unemployment rate of approximately 5% and unemployed adults are excluded from the movement to and from workplace locations and are assumed to stay at home during these periods when other population groups go to school or work.
4. **65 years and older**: seniors are assumed not to work and stay at their home cells during the time the other population groups go to school or work.

### **Distribution of human hosts in the modelled area**

We used cadastral data available from the Queensland government (www.qld.gov.au) that specifies each location in Cairns geographically and is assigned one of 5 types: residential, commercial, industrial, educational or parkland. Households were only assigned to residential properties.

Census data (2011) available from the Australian Bureau of Statistics was used to generate virtual households based on the population age pattern (Figure S1) and household size distribution. The finest spatial resolution for age and household size distribution in Cairns is the Statistical Area Level 1 grade (see Figure S2 B). The best available resolution for total population is the ‘mesh block’ base grade of the Australian census (see Figure S2 A).

Households of a specific SLA1-based size distribution were generated to match the total number of individuals in a mesh block. The so generated households were then geographically distributed according to the Cairns cadastral data (Figure S2 C and D). We assume that one residential property is occupied by exactly one household. The allocation process described above is schematically represented in Figure S3.


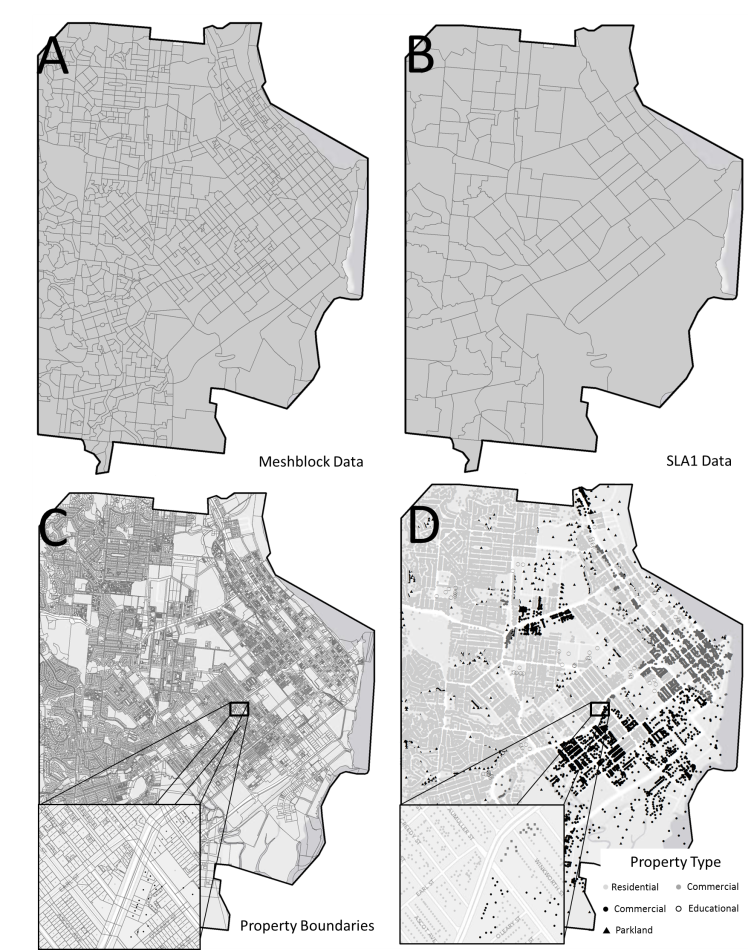


**Figure S2: Available data sources for the generation of households and their distribution. Panel A shows the mesh block spacing of the 2011 census.** Mesh blocks are the census base unit and give information only on the total number of individuals. Panel B shows the statistical level 1 areas (SLA1) of the 2011 census. The SLA1 is the smallest census unit containing all demographic data such as age distribution, and household composition and size. Panel C shows the spatial resolution of the cadastral data available from the Queensland government which lists every property in Cairns (see inset for detail of property boundaries). Panel D illustrates the type of properties as indicated by the cadastral data.


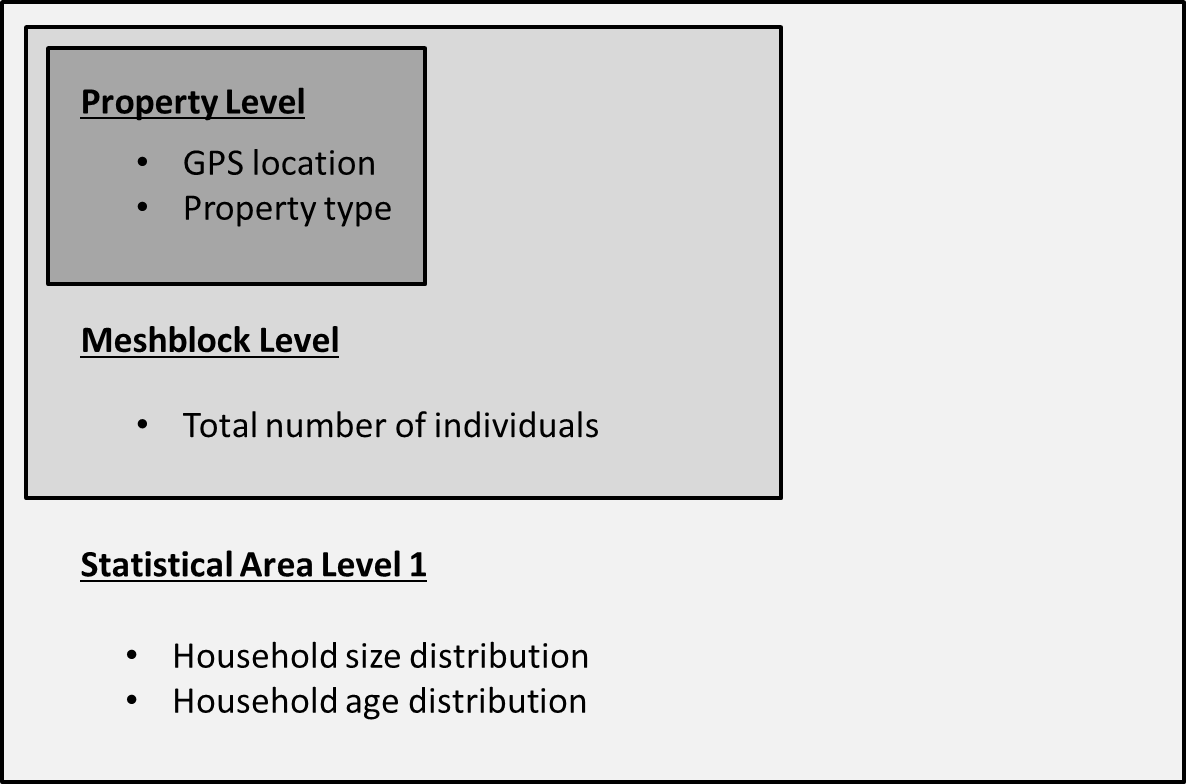


**Figure S3: Schematic showing the process of assembling households.** SLA1 data gave household size and age distribution, mesh block data gave the total number of individuals per area, and the cadastral data (property level) provided the type of property (residential, commercial, industrial etc.) and the exact geographic location.

Once the assignment of individuals to households and households to residential properties was completed, the resulting pattern of households populated with individuals was fused with the 30m x 30m grid based on the geographic household coordinates. For example if two households are geographically located within the boundary of one cell, this cell was assigned as the home cell to the individuals of these two households. This allocation process is schematically presented in Figure S4.

We assume that within a single cell households are not separated further, so mosquito access to each individual in a specific cell is identical.


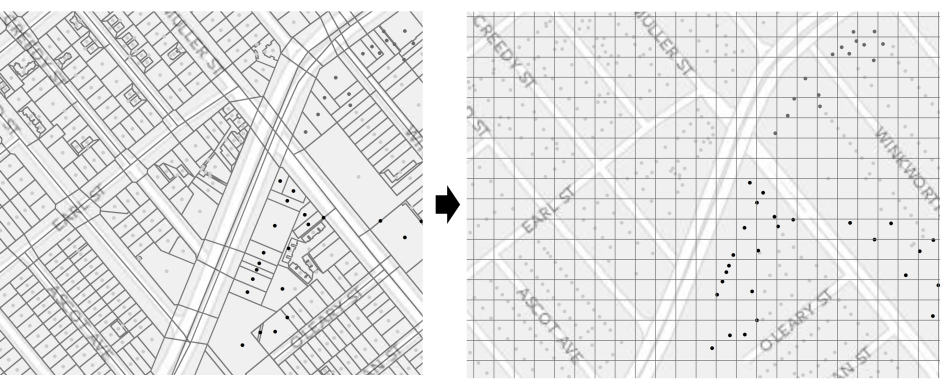


**Figure S4: Final step of allocating households to the square cells, which represent the base unit of the model.** The population of each household (residential property) as well as the other property types enclosed within the boundaries of one cell were combined together with cell properties relating to the mosquito population.

### **Allocation of individuals to workplaces and schools**

Each individual in the working age range (and not assigned as not working) is randomly assigned to a workplace. We assume that there is no distance dependence in the choice for the individual workplaces. The number of people per workplace ranges from 3-8. School children are assigned to the nearest schools and assembled in classes of an appropriate size. Data on school student numbers and class sizes was obtained through the Queensland department of education school register (www.qld.gov.au). Many schools not on the register agreed to provide the respective statistical information when contacted directly.

### **Human Movement**

The model incorporates 2 types of human movement but, of course, real-world movement patterns are much more complex. The features of human movement implemented in the present model are shown in Figure S5.

The first type of human movement is directional and always occurs between 2 specific cells for a given individual. These cells are *i)* the individual’s home cell and *ii)* the individual’s work or school cell. On any given weekday of the modelled period, individuals will go from their home cells (morning) to their work or school cells (daytime) and back to their home cells (evening and night).

The second type of human movement is random and is incorporated in the model to account for humans moving within the modelled area to visit other household locations, go shopping, visit parks etc. This semi-random movement can occur on weekday evenings and during the day and the evening during weekends for all individuals. Based on data from a population survey conducted among European households we used this to estimate the frequency this semi-random human movement to other cells at approximately 4-5 times per week per individual. Human movement in urban areas has been shown to be ranked by distance i.e. the frequency of short trips is higher than the frequency of trips to destinations that are further away. This distance dependence is modelled by applying a γ distribution of distances for the semi-random movement.


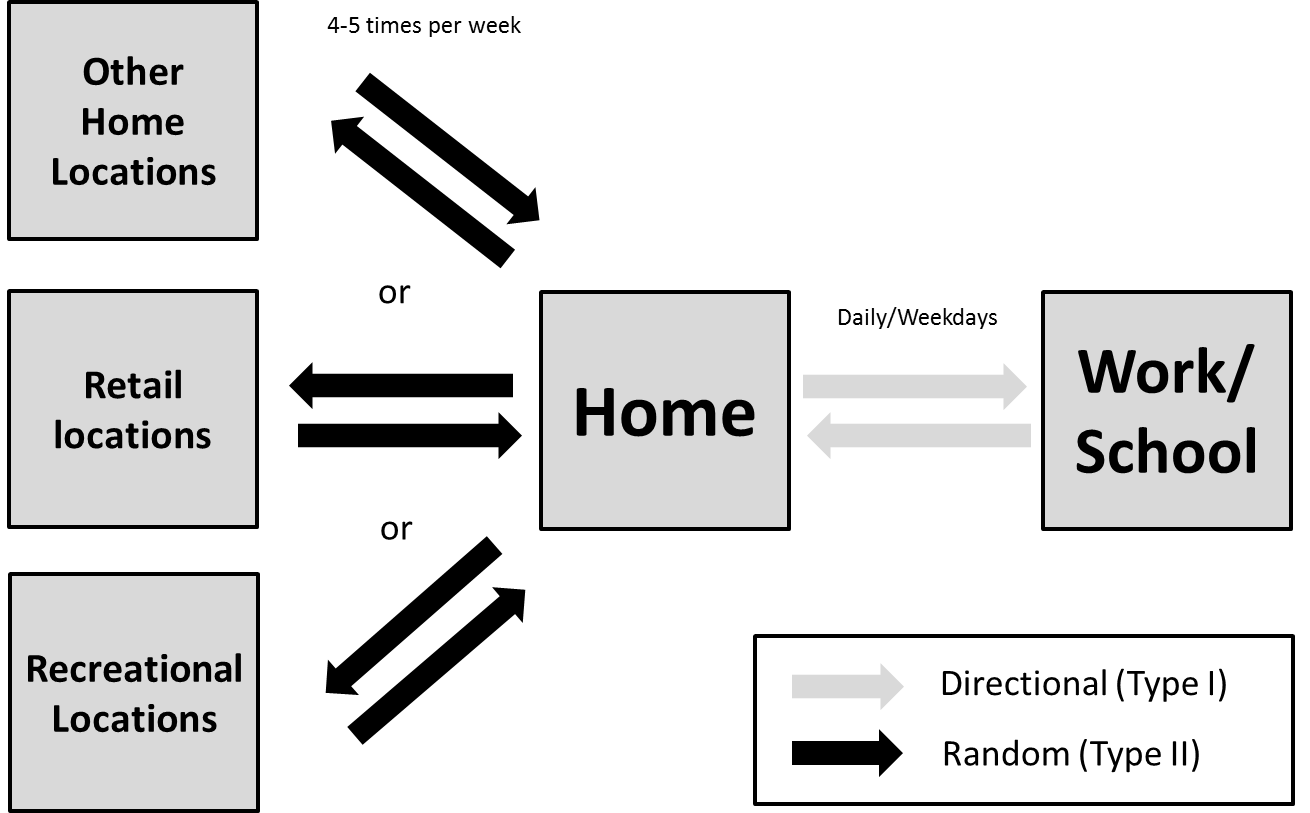


**Figure S5: Schematic representing the different types of cell based human movement.** There are 2 types of movement, i) directional to and from work or school which occurs on every weekday during daytime and ii) random, distance-weighted movement representing all other human movement within the model.

The resulting distribution of distances from home locations for a random sample of individuals from the modelled community on an average day is presented in Figure S6.

**
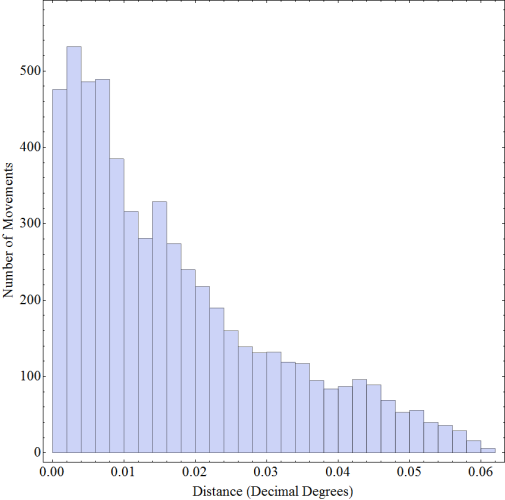
**

**Figure S6: Random human movement distances on an average day for a sample of the modelled population.** Short distances are preferred over long distances based on a γ distribution with shape parameter 1 and scale parameter 0.5.

## Mosquito Population Dynamics Model

### **Overall Model Structure**

The mosquito population dynamics model adopted in this study is similar to that presented by Otero et al. and several others. The model is based on the original work by Focks et al. which is available as the software package CIMSIM and was later put into spatial framework in a software package available as Skeeterbuster.

It should be noted that the model presented here has fewer parameters than CIMSIM or Skeeterbuster and therefore represents a simpler version while still containing all essential stages of the mosquito life cycle. We believe that this approach is appropriate since similarly simplified models such as that of Otero et al. are capable of reproducing observed mosquito population patterns with good accuracy. As was noted by the developers of Skeeterbuster (a spatial version of CIMSIM), many of its detailed aspects, especially the extremely detailed breeding container characterization may be ‘superfluous’, at least for the tasks which we are applying the model in this study.

The core mosquito population dynamics model (only female adult mosquitoes) is schematically shown in Figure S7. It includes **egg (E)**, **larvae (L)**, **pupae (P)** and the two **adult** mosquito populations (**A1** and **A2**) determined by the length of the first versus the lengths of the remaining gonotrophic cycles, with the first gonotrophic cycle being significantly longer than the remaining ones. Adult stages are further subdivided into infection states, as described in the description of the transmission model (page S25).


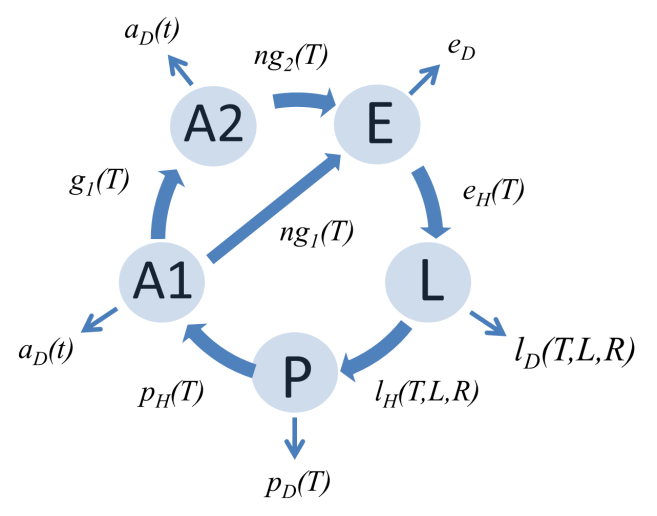


**Figure S7: Model schematic similar to that of Focks 1993, Otero 2006, Yusoff 2011 et al.**: Eggs (E) develop into Larvae (L), Pupae (P), Adults in the first gonotrophic cycle (A1) and adults past the first gonotrophic cycle (A2). Rates of hatching or development are indexed H, death rates are indexed D. *n* is the number of eggs per gonotrophic cycle and g1 and g2 are the lengths of the gonotrophic cycles. Parameters that are functions of other factors are associated with the respective symbols for temperature (T), larvae abundance, (L) Rainfall (R) and time (t).

Bulk immature population numbers (E, L and P) are maintained for each cell. Adult populations (A1 and A2) maintained by tracking individual mosquitoes: adult mosquito objects are created and die stochastically according to a hazard function (Figure S8). This individual-based approach is suitable since it allows arbitrary lifespan and infection state distributions; and because absolute adult mosquito populations in each cell are relatively small (on the order 1-100) the computational complexity is not vastly greater than an equivalent compartmental model.


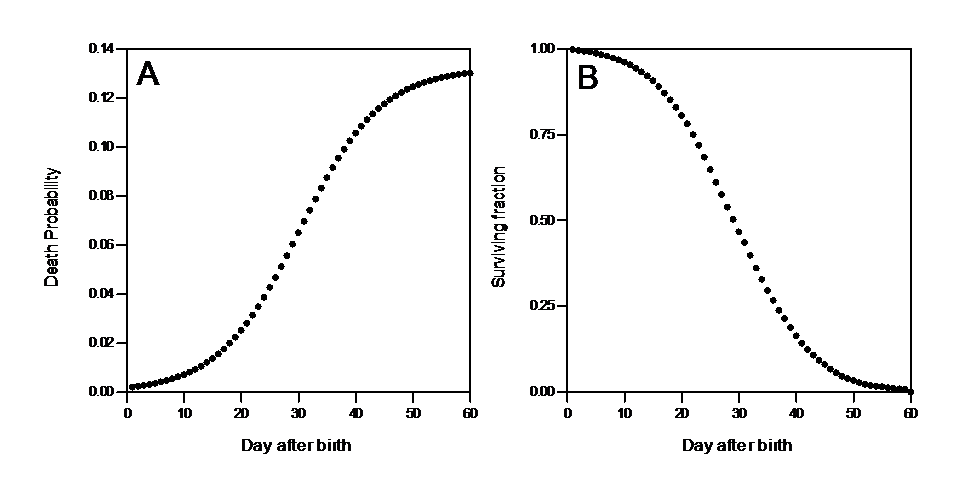
**Figure S8: Mosquito life span.** Panel A shows the hazard function (probability that a mosquito dies based on a binomial trial on a given day). Panel B shows the surviving fraction of mosquitoes, when all mosquitoes are born on day 0.

### **Temperature dependent independent parameters**

The transitions between the mosquito life cycle stages can be grouped into two types of parameters, those that are assumed to be temperature dependent and those that are assumed to be constant. A variety of prior dengue modelling studies have adopted the same assumptions about the temperature dependent parameters that were first described by Focks et al. (, Table 2, or the equivalent Table in ). In this study the same approach was utilized, based on the same enzyme kinetics model as developed by Sharpe and refined by Schoolfield as used by CIMSIM, Skeeterbuster, the similar model developed by Otero and those of others. We do not present the same table and equations again, as they can be found in Focks 1993, Otero, 2006 and Yusoff 2011.

The temperature dependent parameters are (compare to schematic representation in Figure S7): *i)* **egg development** (e_H_), *ii)* **larvae development** (l_H_), *iii)* **larvae mortality** (l_D_), iv) **pupae development** (p_H_), *v)* **pupae mortality** (p_D_) and the *vi)* **gonotrophic cycle durations** for Adults 1 and 2 (a1_H_ and a2_H_). We thus assume that these parameters are predetermined and do not use them in the calibration process of the model to observed mosquito data.

The temperature independent constants in the model are (compare to schematic representation in Figure S7): *i)* **number of eggs** laid per gonotrophic cycle (n=63), ii) **ideal egg mortality** (e_D_= 0.011 per day) and iii) adult mortality, which is based on an age dependent hazard function (Figure S8) as in Chao et al. The values assigned to these parameters are based on previous estimates and are given in parentheses.

The predefined parameters used in the mosquito population dynamics model are shown in Table S 1.

### **Adult mosquito death**

Adult mosquitoes are assumed to die according to a hazard function similar to that used in , and based on . Probability of death is dependent on a logistic function in the form of Equation S1,

$H(t)=\frac{ae^{bt}}{1+\frac{as}{b}(e^{bt}-1)}$ (S1)

with parameters a = 0:0018, b = 0:1416, s = 1:0730 . The time dependent probabilities of death and the resulting adult mosquito life-span distribution at any given time of the simulation are shown in Figure S8. Maximum mosquito age was set to 60 days as in .

### **Rainfall and evaporation dependent parameters**

***Cell capacity to sustain mosquito larvae***

In contrast to Otero et al. , we assume that mosquito breeding sites appear and disappear with the ambient availability of water. This is necessary since Cairns has very distinct wet and dry seasons, and mosquito breeding sites show a significant decrease during the dry season. In contrast to the detailed CIMSIM and Skeeterbuster approach where the characteristics of each potential container that may serve as a breeding site has to be determined, we developed a simpler approach. Instead of characterizing single containers and their specific properties, we introduce a more abstract value of a cell’s **capacity to sustain mosquito larvae**. Each 30 x 30 m cell in the model area is assigned a maximum capacity to sustain mosquito larvae, based on its properties. The process of assigning the maximum capacity to sustain larvae to each cell is described below. We assume that a cell’s maximum capacity to sustain a larvae population (*L_max_*) is only reached when the availability of water is high. If the availability of water is limited, a cell will have a lower capacity to sustain larvae (*L_Real_*). While *L_max_* and *L_Real_* are local, cell-specific properties we estimated the availability of water or the ‘water level’ (*W_Real_*) globally, based on rainfall and evaporation records for the desired modelling period. We assumed that the water level fluctuates between a minimum (*W_min_*) and a maximum (*W_max_*) boundary level. The minimum level represents e.g., artificially watered breeding sites and standing water shielded from evaporation. The maximum level indicates an upper limit above which potential breeding sites can be imagined to ‘overflow’. To calculate the daily change in water level we add rainfall and subtract evaporation (both available in mm at the weather data archive of the Australian Bureau of Meteorology, www.bom.gov.au). If the water level is above the designated maximum, further rainfall does not add to the water level as the breeding sites are overflowing. If the calculated water level is below the minimum, no further reduction due to evaporation is allowed. This approach can be described through the following conditional equations (S2) in which RF and EV stand for rainfall and evaporation, respectively:

IF *W*_Real_ + *RF* – *EV* > *W*_max_ then *W*_Real+1_ = *W*_Real_ – *EV*

IF *W*_min_ < *W*_Real_ *RF* – *EV* < *W*_max_ then *W*_Real+1_ = *W*_Real_ + *RF* – *EV* (S2)

IF *W*_Real_ + *RF* – *EV* < *W*_min_ then *W*_Real+1_ = *W*_Real_ + *RF*

An exemplary water level (*W_Real_*) calculation based on rainfall and evaporation data from Cairns from 2006 to 2008 is shown in Figure S9.


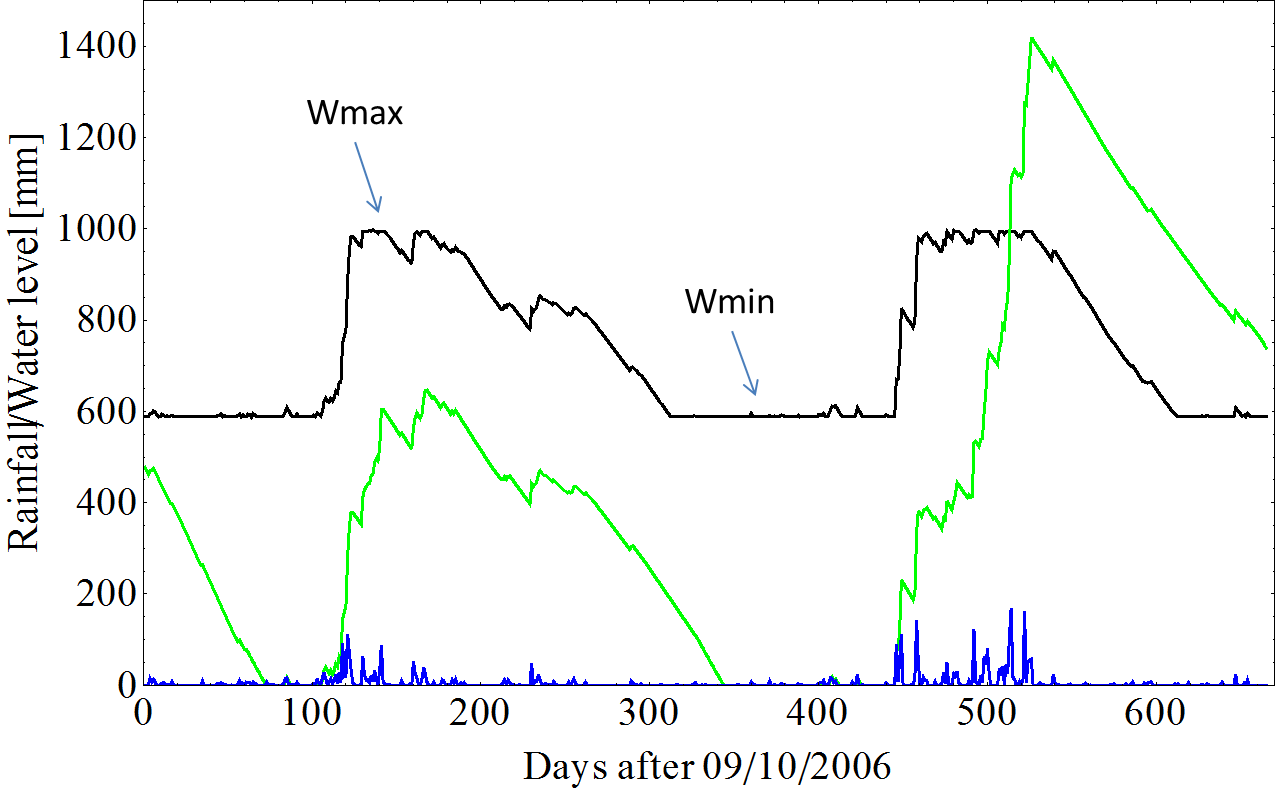


**Figure S9: Rainfall (blue) and resulting water level (black) based on the daily rainfall and evaporation data in Cairns 2006-2008.** The green curve shows the water level as it would be without limiting it to between *W_min_* and *W_max_*.

We have assumed that the capacity of a cell to sustain a mosquito larvae population (*L_Real_*) scales linearly with the water level *W_Real_* until it reaches the cell specific maximum capacity L_max_. This dependency is expressed using Equation S3

$L_{\text{Real}}=L_{Max}\times\frac{W_{\text{Real}}}{W_{\max}}$ (S3)

The equation causes *L_Real_* to approach *L_max_* when *W_Real_*~ *W_max_* and approach *L_Max_W_min_*/*W_max_* when *W_real_*~ *W_min_*. While some previous mosquito population dynamics models do not account for rainfall patterns at all , the approach presented here is simpler than the ones used by CIMSIM and SkeeterBuster. .

***Water level dependent egg hatching***

While the temperature dependence of the egg hatching rate (*e_H_*) is maintained, we also assumed that it is dependent on water availability. If the water level is low, eggs hatch with a reduced rate and as water level increases due to rainfall, eggs hatch at an increased (ideal) rate similar to that given in . We assume that even a small amount of rain leads to an increase nearly to the ideal hatching rate, thus ‘kick-starting’ the mosquito population. This quick response to rainfall can be modelled with a Hill function as shown in Equation S4.

$e_{H}=(e_{H,Ideal}(T)+e_{H,\min}(T))\times\frac{a\left(\frac{W_{\mathrm{Re}al}}{W_{\max}}\right)^{h}}{1+a\left(\frac{W_{\mathrm{Re}al}}{W_{\max}}\right)^{h}}+e_{H,\min}$ with (S4)

As the minimum egg hatching rate is unknown we assume it to be fraction *r* of the ideal rate which we will later adjust to calibrate the model to the observed mosquito data. The exact values of *a* and *h*, which determine the shape of the Hill function are also unknown, however we can speculate that egg hatching is triggered readily even by limited rainfall events. We thus choose *a* and *h* to have relatively high values, so that *e_H_* tends towards *e_H,ideal_* even with a small increase in the water availability (*W_real_*). We do not assume any further constraint in egg hatching since mosquito eggs are independent of external food sources. The response pattern of *e_H_* to *W_Real_* is shown in Figure S10.


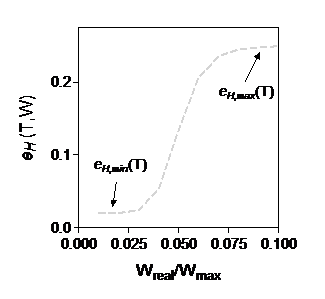


**Figure S10: Egg hatching rate dependence on water availability.** It is assumed here that the egg hatching rate is highly responsive to the onset of rainfall at the end of the dry season and only small changes in the water availability result in expansion of *e_H_* to its ideal value. Therefore appropriate Hill parameters a = 20 and h = 8 are chosen.

***Heavy Rain***

We assume that heavy rain has a negative impact on immature mosquito stages as they will be washed out from some breeding sites such as sump pits [28]. This is an effect that is only enabled when it rains heavily and water levels close to *W_max_* are reached. When enabled, a fraction ω_0_ of eggs, larvae and pupae will be washed out on each day. Again we realize this using a Hill function with rigid response parameters *h* = 20, *a* = 1.2, meaning that only when *W_max_* is approached, washout will have an effect. The equation is:

$\omega=\omega_{0}\times\frac{a\left(\frac{W_{\mathrm{Re}al}}{W_{\max}}\right)^{h}}{1+a\left(\frac{W_{\mathrm{Re}al}}{W_{\max}}\right)^{h}}$ (S5)

The resulting dependency of *w* on *W_Real_* is shown in the figure below (Figure S11).


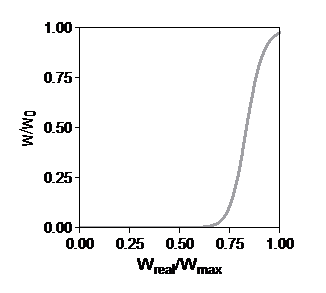


**Figure S11: Fraction of immature stages being ‘washed out’ of breeding sites due to heavy rain.** The ‘washout’ only has an effect when *W_real_* is close to *W_max_.*

### **Population density dependent larval parameters**

We make similar assumptions as previous studies on the density dependent growth of larvae. . Without density dependent larvae inhibition, the resulting mosquito numbers would increase exponentially, making these limitations essential to the model. Measures of emergence of pupae from field containers suggest that density dependence is an important factor regulating *Ae. aegypti* production in Cairns [28]. As in previously published work, we assume that both larvae development and death are density dependent. Density limitation enters the model through the assumption that a breeding site can hold only a specific number of mosquito larvae (we called this the ‘ambient capacity of a cell to sustain larvae’ or *L_real_*). *L_real_*, is dependent on the availability of water as described above. If the larvae density (*L*) in a cell approaches or exceeds the ambient capacity *L_real_*, mosquito larvae develop more slowly and die more rapidly due to food limitations and increased predation . While we adopt this same reasoning as previous work, we implement it in the model in a slightly different way.

***Larvae Development***

Under ideal conditions larvae are assumed to develop with a rate *l_H,Ideal_* a maximum value that is only constrained by temperature. As the larvae density (*L*) increases towards the ambient capacity *L_Real_*, this ideal development rate will decrease until it approaches a minimum value *l_Hmin._* In contrast to previously used quadratic or linear functions, we believe that a sigmoidal Hill type equation can be used to describe this process.

$l_{H}=(l_{H,Ideal}(T)-l_{H,\min}(T))\times\frac{a\left(\frac{L}{L_{\text{Real}}(R)}\right)^{-h}}{1+a\left(\frac{L}{L_{\text{Real}}(R)}\right)^{-h}}+l_{H,\min}(T)$ with $l_{H,\min}(T)=q\times l_{H,ideal}(T)$ (S6)

As the value for *l_H,min_* is unknown we therefore assume that it is a fraction *q* of *l_H,ideal_* which will later be adjusted to calibrate the model using available mosquito data. The exact values for *a* and *h* are also unknown*,* but we assume the response of *l_H_* to a growth in the larvae population (*L)* to be relatively slow, in contrast to the egg hatching that can be triggered by a small rainfall event. We therefore choose *a* and *h* to low values (*a* = 2, *h* = 1).

The resulting curve of *l_H_* over *L/L_real_* is shown in Figure S12. Note that this larval density dependent growth rate *l_H_* still incorporates temperature dependence (not depicted here).


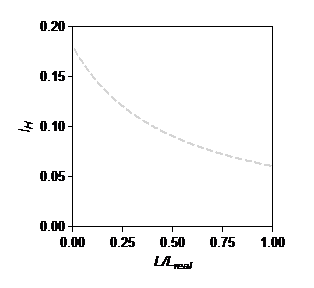


**Figure S12: Density dependent larvae development rate.** It is assumed that larvae develop at an ideal (temperature dependent) level, when larvae density (*L/L_real_*) is low. The larvae development rate decreases as larvae density increases. We assume this decrease to be a gradual effect and choose appropriate Hill parameters *a* = 2, *h* = 1 to reflect this.

Previous work, e.g, Otero et al. modelled density dependent larvae development inhibition using a slightly different, quadratic equation (in the form of *αL(L-1)*). We assume that an equation which can be constrained at both ends to a minimum and maximum (such as the Hill function) may be more appropriate, however since the real kinetics involved are unknown both approaches are somewhat speculative

***Larvae Death***

We also assume that larvae (*L*) die at a higher rate when their density is high. This may be due to nutritional constraints or increased predation with density, as noted previously . Under ideal conditions, larvae die at a minimum rate *l_D,min_*. which is purely dependent on temperature. As larvae number *L* approaches or exceeds the current capacity *L_Real_* this death rate increases to *l_D,max_*. Also here, we adopt the general pattern of a Hill type equation allowing a sigmoidal increase from *l_D,ideal_* to *l_D,max_* as shown in equation S7.

with (S7)

Again, the maximum death rate *l_D,max_* is unknown, therefore we assume it to be is a value between *l_D,ideal_* and 1 given by a multplier *k*. Later, we will use *k* to fit the observed mosquito data to the model. As the exact kinetics of increased larvae death with larvae density are also unknown, but since it can be assumed that the response of *l_D_* to an increased larvae population (*L)* is relatively slow, we choose the paramters *a* and *h* that govern the shape of the Hill function to assume relatively low values (*a* = 2, *h* = 4). The resulting dependence of *l_D_* on *L/L_real_* is shown in the figure below (Figure S13).


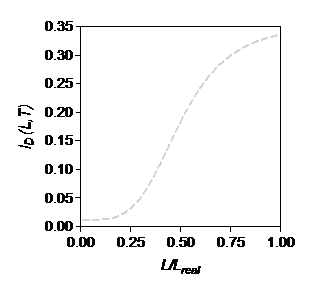


**Figure S13: Density dependent Larvae death.** Previous work (Otero et al. ) has modelled larvae density dependent larvae death as a step-wise function (with a low value for low larvae densities and a high value for high larvae densities). We chose a slightly different approach, assuming a sigmoidal relationship between larvae death and larvae density.

While Otero et al. used a simple step function to model density dependent larvae death , we use a Hill equation as a simple model to describe density dependent population growth. Although it is normally used in biochemistry to model saturation binding, hill type equations have been used in previous models of density dependent population dynamics. As described in the previous paragraphs, we make assumptions on the shape of the Hill functions. Table S2 summarizes the Hill shape parameters chosen in the present study.

### **Sensitivity analysis in the Hill parameter space**

Since we do not know the exact values for the parameters used in the Hill type equations it is necessary to conduct a sensitivity analysis exploring the parameter space in a relatively wide margin around the chosen parameters (which we now aim to keep fixed during the fitting process). High sensitivity of the overall predicted mosquito density patterns should not be present, i.e. even when significantly varying the values presented in Table S2, the resulting predicted relative temporal mosquito patterns should all belong to a scalable family of curves. To test this assumption, we create a parameter space by varying each of the parameters in Table 1 by +/-50% of its value. We now apply the model to 1000 random samples of this parameter space, keeping all other model parameters fixed at their fitted or prescribed values. The resulting family of curves is shown in the Figure S14 below.


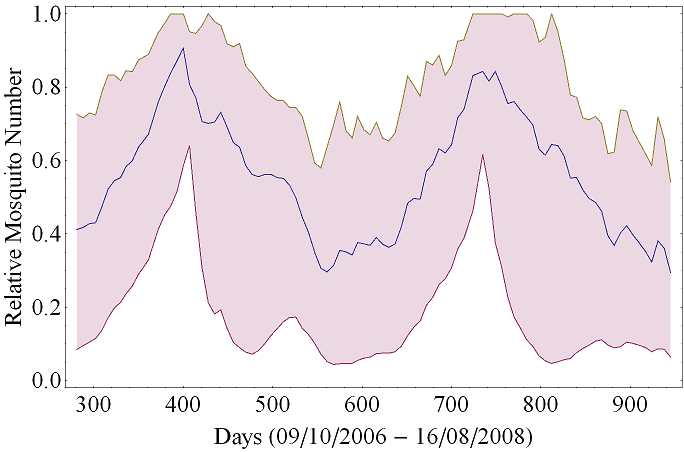


**Figure S14: Sensitivity analysis of the Hill equation parameter space**. Although there are fluctuations in the predictions depending on the choice of the Hill parameters (a,h), the overall pattern of relative mosquito abundance remains similar.

Figure S14 shows that although the choice of the parameter values for the parameters given in Table 2 do impact on the predicted curves, all members of the resulting family of curves are still amenable to the observed mosquito pattern (Figure S22, black line). This suggests that variation of other parameters will still result in an acceptable fit regardless of the exact choice of the Hill parameters.

### **Spatial Heterogeneity of Breeding Sites**

***Derivation of cell specific breeding site abundance***

This section explains the derivation of the cell specific maximum larvae population (L_max_), which is the cell specific parameter that introduces spatial heterogeneity into the mosquito population dynamics model. Heterogeneity also enters the model in other ways, e.g. through mosquito flight, however with a much less pronounced effect. In the present study we aimed to tie L_max_ to cell specific geographic and demographic features. Several previous spatial modelling studies of *Aedes* population dynamics assume that the number of mosquito breeding sites is a function of the number of dwellings i.e. there is a specific fixed number of breeding sites (and thus capacity to sustain mosquito larvae) per house). . Classic compartmental models of vector borne disease transmission often use a mosquito per human ratio. Skeeterbuster is the most detailed spatial model for *Aedes* dynamics and requires the input of specific breeding ‘container’ characteristics which are only obtainable through extensive field surveys. Although a detailed classification of breeding habitats (through container surveys) may result in more detailed data, it is questionable whether it is feasible to collect such data for a modelling area the size of the one used here; and even if collected whether, for our purposes, it would significantly improve the accuracy of the model. We therefore utilised satellite imaging and demographic data to derive spatial information about mosquito habitats.

In the present study we assumed two cell specific geographic features to be positively correlated with a cell’s capacity to sustain mosquito larvae *i)* degree of vegetation in a cell and *ii)* number of dwellings per cell. We base this assumption on previous work and the positive associations found between mosquito trapping data from Cairns and these two characteristics found in the course of this study. We assume that the number of dwellings per cell is more important than vegetation, since it provides *i)* humans which are the source for blood feeding and *ii)* human-made breeding habitats such as rainwater tanks, underground sumps, flower pots etc. However, we assume that vegetation adds an additional benefit to a cells capacity to sustain larvae, since it may prevent increased solar exposure (e.g, resting places in foliage) and lower local rates of evaporation. In addition, vegetation features can provide additional breeding sites such as fallen palm tree leaves. Previous studies have shown a highly significant correlation between vegetation cover and the abundance of *Aedes aegypti* breeding sites. We introduce a *‘breeding site abundance index (B)’* and suggest a simple dependence between *B*, the number of dwellings (*D*) and vegetation cover (*V*) in the form of:

*B=D+DV+B_min_* (S8)

We furthermore assume a minimum breeding site index *B_min_* to be present independently of the cover with houses and vegetation. The model structure also allows to account for special sites like large underground sumps etc. by entering a *B* value for a cell manually, however we do not use this feature in the present study. Figure S15 shows the distribution of *‘breeding site abundance indices (B)’* based on Equation (S8). We normalize B to fall between 0 and 1 since it is only a relative value that stands for the suitability of a cell to allow mosquito reproduction. We believe that the general distribution of expected mosquito breeding sites is reasonably well represented by the resulting pattern. For example, industrial areas have a low breeding site index (blue). Houses with significant vegetation cover surrounding them as e.g, in Parramatta Park have a high breeding site index (red).


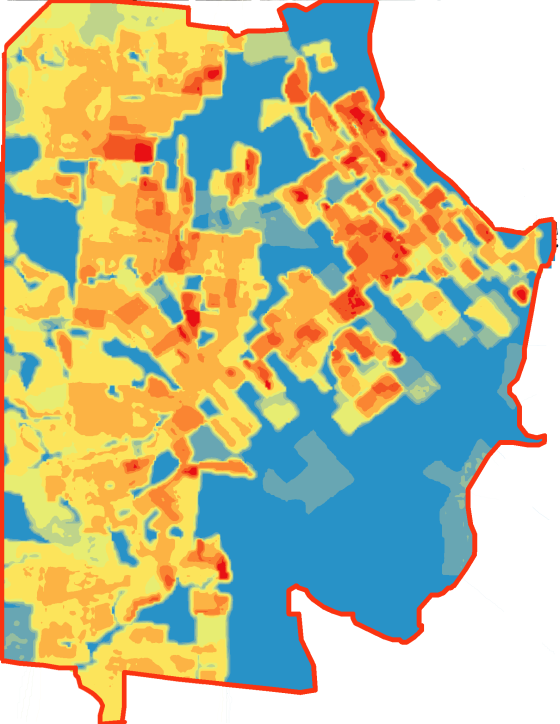


**Figure S15: Spatial distribution of breeding site indices.** The colour coding is: blue: low: 0 - 0.1, orange: medium: 0.3 - 0.6, red: high: 0.6 - 1.

***Validation of Mosquito Habitat Heterogeneity with Trapping Data***

Since the process of assigning a mosquito breeding site abundance index to each cell as described in the previous section does not rely on fitting to the data, we aim to validate it using available trapping data (shown in Figure S16). For this purpose available trapping data from Cairns for the years 2006-2013 was used. These data were kindly provided by Scott Ritchie (James Cook University) and Peter Cook (Monash University). We rely on the data collected using commercially available BG traps since these are considered to produce more reliable mosquito density estimates [28]. However, the available data are limited to a small proportion of the modelled area.


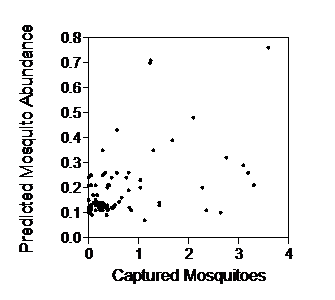


**Figure S16: Correlation between the average number of trapped mosquitoes per trap per day and the predicted mosquito abundance index.** Although there is a highly significant correlation, the coefficient of determination is not very high.

To validate the assignment of breeding site abundance indices using the trapping data we assume that where there are high numbers of trapped adult female mosquitoes, breeding conditions are good i.e. there is a positive correlation between the number of trapped mosquitoes in a cell and the cell’s specific breeding site abundance index.

To test this hypothesis, we plot the predicted ‘breeding site abundance index’ against the observed average number of mosquitoes trapped per day in the same cell. The resulting correlation highly is significant (Pearson p<0.0001) but not strong (Pearson R=0.48).

We conclude that the data does not allow for derivation of a continuous spectrum of breeding site abundance indices as shown in Figure S15 *i)* because the there is significant scatter and the correlation coefficient (R=0.48) is weak and *ii)* trapping data is limited to certain regions in the model area (see Figure S18 for trap locations).

We therefore resort to ranking the data into a number of distinct groups which are significantly different. The available data only permits the establishment of 2 such groups, namely *i)* trapped mosquito numbers below 1 per trap per day and *ii)* trapped mosquito numbers above 1 per trap per day as shown in Figure S17.


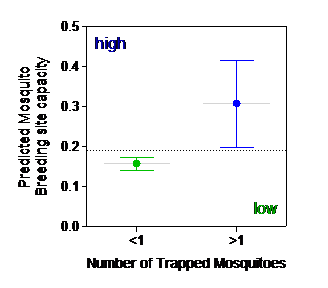


**Figure S17: Ranking of breeding site abundance indices according to the number of trapped mosquitoes per day**. Separation of the data into two groups results in two clearly distinct populations. We therefore conclude that a classification of the model area into cells with low and cells with high breeding site abundance indices is well supported by the data.

The difference between these two groups is highly significant and the traps assigned to each of the 2 groups yielded the group specific number of mosquitos with 95% confidence i.e., very few traps are located in the wrong category.

A map of the resulting two ‘classes’ of mosquito densities (low/high) is shown in Figure S18.


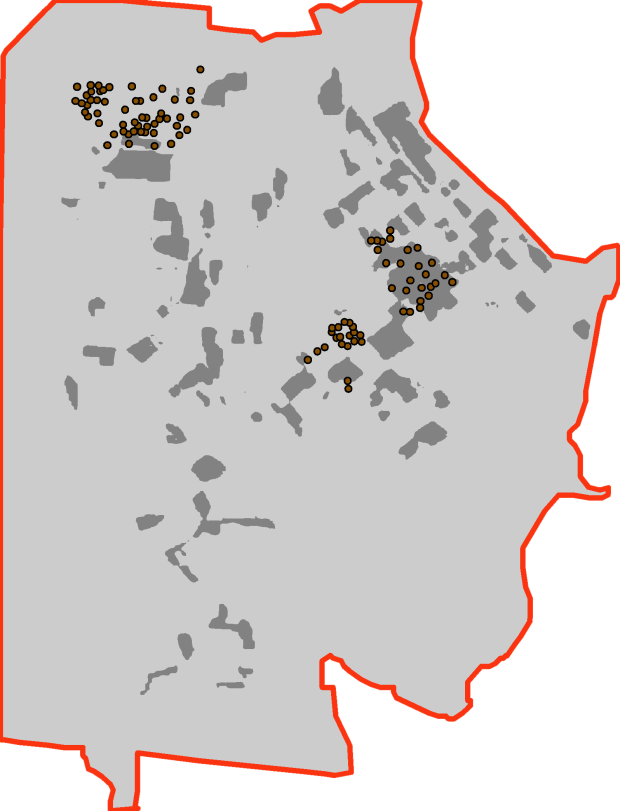


**Figure S18: Classification into low vs. high breeding site density areas.** The light grey areas are assigned to the low breeding site index class (class 1) and the dark grey areas are assigned to the high breeding site index class (class 2). The markers indicate the trap locations.

The classification also indicates the magnitude difference between the low and high mosquito breeding site abundance classes. Based on Figure S17, the mosquito density in high breeding site abundance index cells should be approximately 2 fold of that in low breeding site abundance index cells.

The breeding site abundance index is only a measure of relative mosquito abundance. The choice of the *cell capacity to sustain larvae (L_max_)* parameter for each of the two classes will determine the absolute mosquito numbers. The calibration process using trapping data to obtain these class specific L_max_ values is described on page S22 to S23.

### **Adult mosquito flight**

We assume that adult mosquitoes (stages A1 and A2) can fly to neighbouring cells in the mornings and the evenings (time steps 1 and 3 of a model cycle) and flight direction is completely random. The only parameter that determines mosquito flight is the probability *f* that a mosquito flies into a neighbouring cell. Data presented from mark-release studies with *Aedes aegypti* in Cairns was used to estimate a suitable value for *f*. . Russell, Webb et al. estimated that approximately 25 % of mosquitoes fly outside a 100 m radius from the release point within 15 days of the release whereas 75% stay within the 100 m radius. We adjusted *f* using simulated mark-release experiments as shown in Figure S19 to reflect this observation. As a result of these simulations we set *f* to 0.25, i.e., a 25% probability that a mosquito flies to another cell on either a morning or evening time step of the model. This resulted in a good approximation of the observations made by Russell, Webb et al.

###
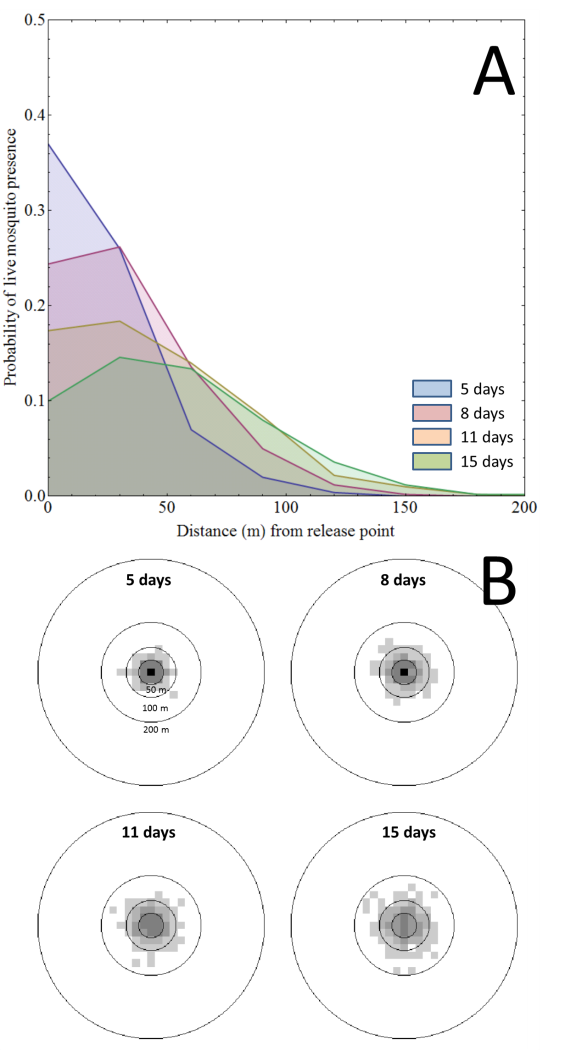


**Figure S19: Exemplary mosquito dispersion simulation.** Panel A: Probability that a living mosquito is found at a certain distance from the release point a certain number of days after the release of 500 mosquitoes in one location. Panel B: Schematic representation of the spatial mosquito spread. Mosquito number ranges are indicated by the grey shading (e.g., black cells contain more than 20 mosquitoes, dark grey cells 5-19 mosquitoes and light grey cells 1-4 mosquitoes). The concentric circles indicate distance from the release point of 50m, 100m, 200m and 450m respectively. Previously quantified localized spread of infections as well as mosquito mark-release data were used to calibrate mosquito spread such that after 15 days ~25% of mosquitoes would be found outside a 100m radius from the release point. [9,37] Each grid tile is equivalent to an area of 30 x 30 m. Mosquito life-span is was set to an average 18 days based on the hazard function in Figure S8.

### **Fitting the Mosquito Population Dynamics Model**

***Input Data***

In order to validate the model and obtain values for unknown parameters, the mosquito population dynamics part of the model was calibrated using an observed mosquito density pattern as shown in Figure S20.


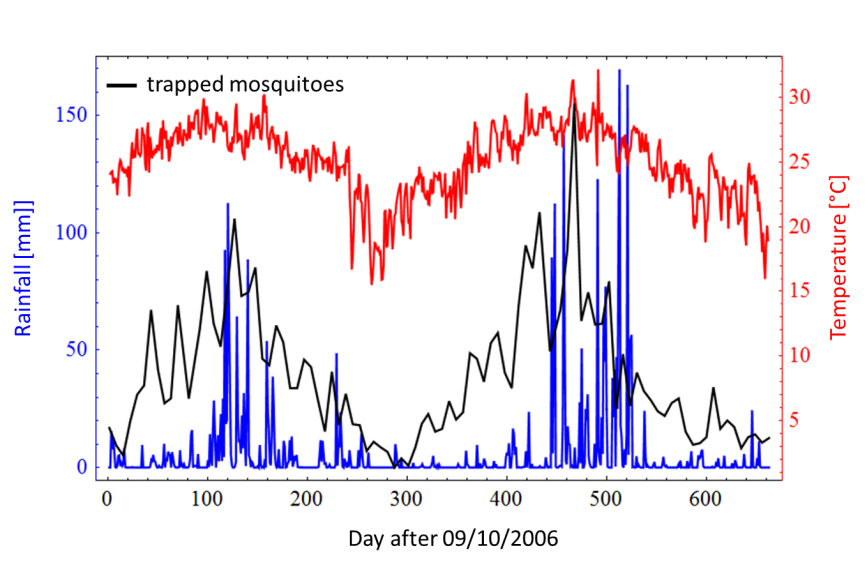


**Figure S20: Rainfall (blue), temperature (red) and average number of trapped mosquitoes per trap per day (black) data from 09/10/2006 to 16/08/2008.** Note that mosquito trapping data is presented without a scale, as the absolute mosquito density is unknown and the trapping data only indicates the relative mosquito density pattern.

The data shown in Figure S20 is for the time between 09/10/2006 and 16/08/2008. During this time, no major dengue outbreaks were recorded in the modelled area, so that it is expected that anthropogenic mosquito control did not significantly affect the mosquito population. Mosquito trapping data is shown without a scale, since the absolute number of mosquitoes is unknown. However, the calibration process was aimed at obtaining average adult female mosquito numbers in the range of 8-24 mosquitoes per property, as this has been determined for Cairns by various previous studies.

***Temperature dependent Parameters***

As noted in the mosquito population dynamics model description, all temperature dependent parameters are predetermined by the temperature profile and the methods laid out in detail in and . Figure S21 shows the profiles for all temperature dependent parameters for the weather from 09/10/2006 to 16/08/2008 (Figure S20)


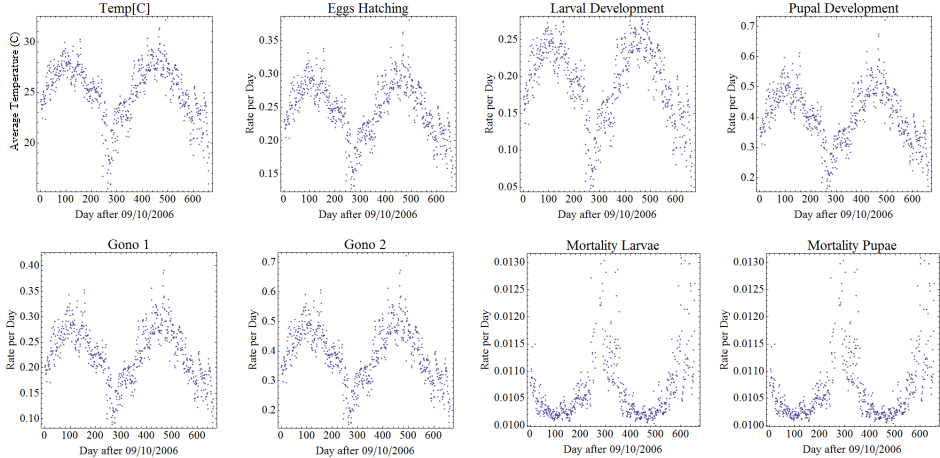


**Figure S21: Temperature dependent parameters for the time from 09/10/2006 to 16/08/2008.**

The temperature independent parameters are kept fixed to the values given in Table S1. The kinetic Hill parameters are kept fixed to their respective values as given in Table S2, which were chosen according to the rationale explained previously.


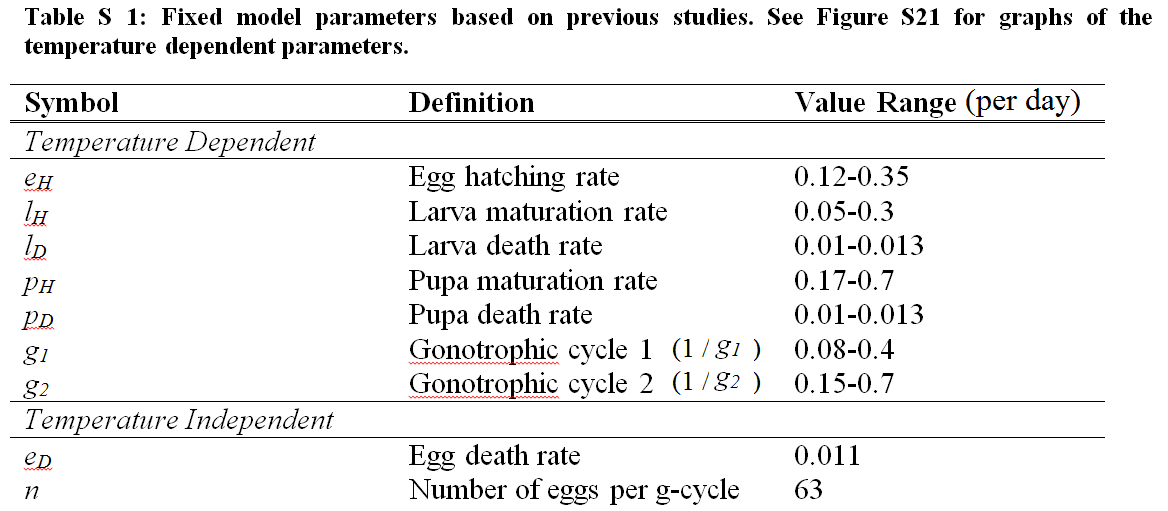


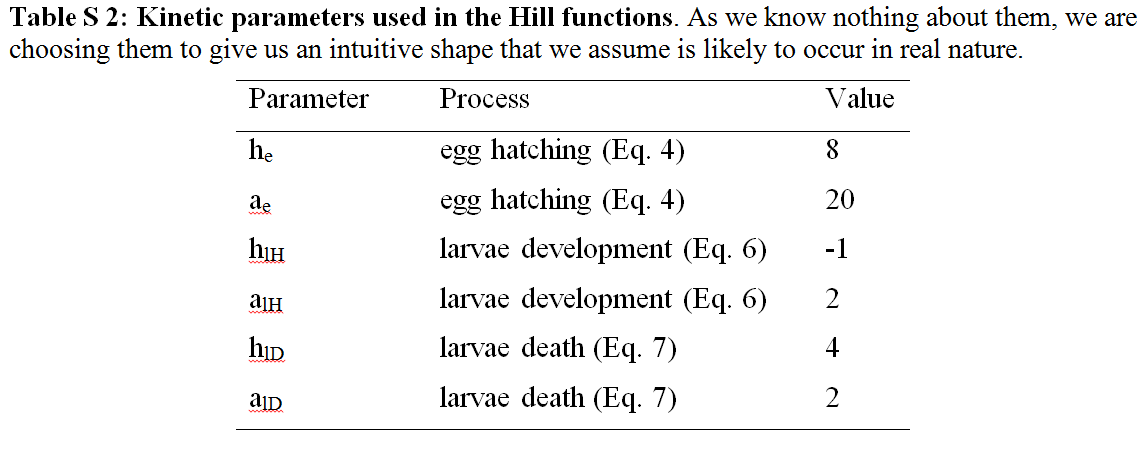


This still leaves a range of parameters that can be varied to achieve a fit to the data. These are:

1. the *ratio of minimum to maximum water level*
2. the *minimum density dependent larvae development rate* as a function of the ideal larvae development rate (see equation S3)
3. the *maximum density dependent larvae death rate* as a function of the ideal mosquito death rate (see equation S4)
4. the *minimum water level dependent egg development rate* as a function of the ideal egg development rate (see equation S5)
5. the *maximum heavy rain washout fraction* *w_0_*

The most important of these parameters is *i)* as the model is highly sensitive to changes to the water level ratio. Since the mosquito population dynamics model runs separately in every cell of the model area, we calculated the sum of all cells and fitted it to the overall mosquito abundance pattern. The model was calibrated to the relative mosquito pattern. Since the maximum capacity for a cell to sustain mosquito larvae (*L_max_*) will only affect the overall scaling (absolute mosquito numbers) but not the relative mosquito abundance pattern we can assign any value to *L_max_*, keeping the ratio between the two breeding site abundance index cell classes fixed at a value of *n* (*L_max,Class1_* = *nL_max,Class2_*). The ratio *(n)* of L_max,Class1_  and L_max,Class2_ was chosen such that the resulting adult mosquito numbers were 2 fold higher in the breeding site abundance index class 2 cells. Variation of L*_max_* can now be used to scale to the absolute mosquito number.


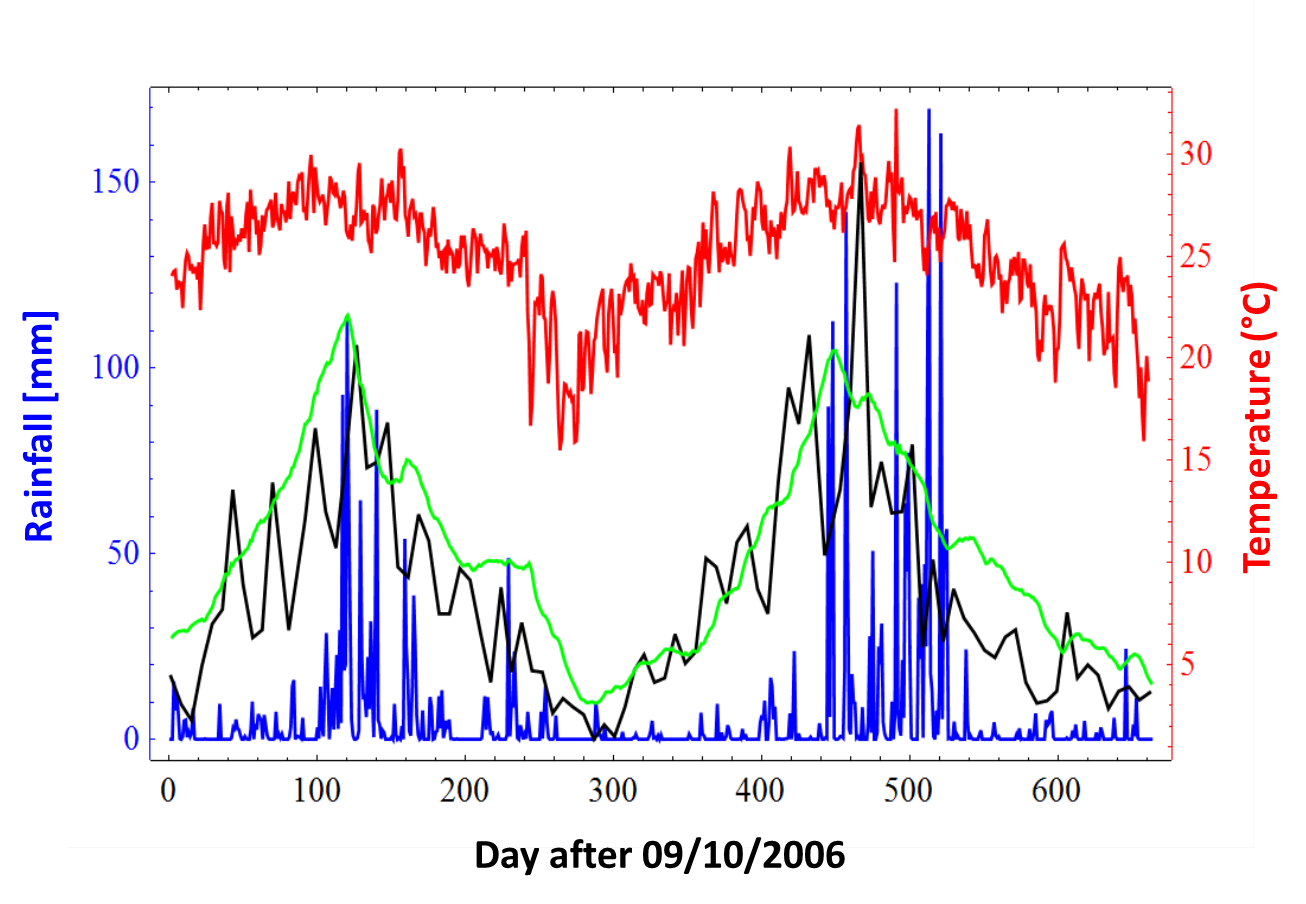


**Figure S 22: Fitted Mosquito Density (green) versus observed density (black).**

We now generate a sufficiently large sampling of the parameter space of *i)-v* (>10^6^ random combinations of parameters *i-v*) and fit to the **relative** observed mosquito trapping data (presented in Figure S20) using a ‘random walk’ type fitting approach. The resulting calibration is shown below.

We can now use *L_max_* – the maximum cell capacity to sustain a larvae population, to scale the absolute mosquito population. This approach is based on our assumption that all modelled cells should follow a similar mosquito density pattern, yet some will be more suitable for mosquito reproduction than others. We scale *L_max_* (*L_maxClass 1_* and *L_max, Class 2_=nL_max,Class 1_*) to result in an overall curve that reflects an average mosquito number of 8-24 mosquitoes per dwelling. Since our model area comprises approximately 28,000 dwellings, we aim to adjust *L_max_* to obtain an average mosquito number of around 2.24 x 10^5^ to 6.72 x10^5^ mosquitoes in the modelling area. A resulting graph showing absolute mosquito abundance is shown in Figure S23.


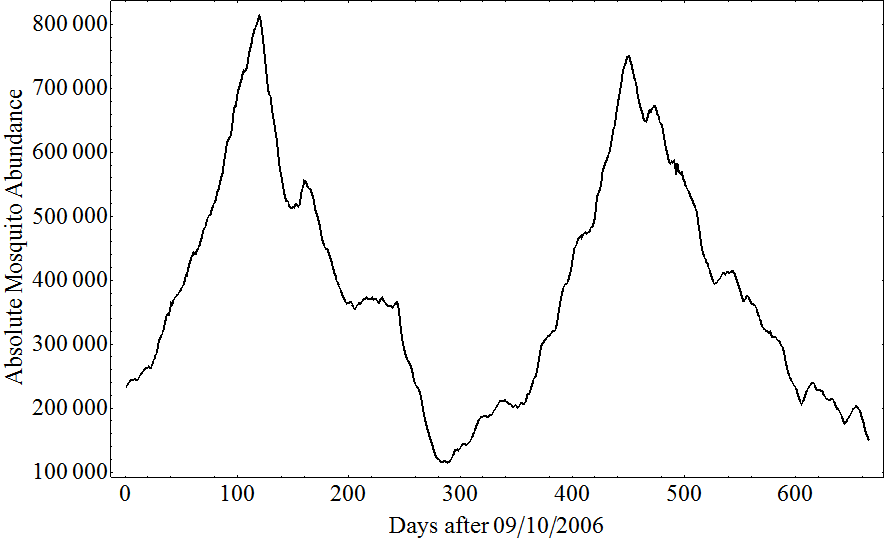


**Figure S23: Absolute mosquito abundance in the model area based on 8-24 mosquitoes per dwelling.** Note that we further slightly adjust the absolute number of mosquitoes via *L_max_* when calibrating to the unmitigated phase of the 2003 dengue outbreak.

The calibrated version mosquito population dynamics model allows for the calculation of temperature and rainfall dependent mosquito profiles for other years. It should be noted that large dengue fever outbreaks and the resulting vector control measures will impact on the mosquito population beyond the scope of what can be fitted with the pure mosquito population dynamics model. Only the coupled overall mosquito population dynamics/ dengue transmission and dengue control model can be used to estimate mosquito population curves in years with large dengue outbreaks.

## Virus transmission model

This study uses the classic SEIR transmission model used in other studies, including those modelling dengue. There are two components to the transmission model *i)* infection in humans and *ii)* infection in mosquitoes.

### **Infection and virus incubation for humans**

In the model humans can assume one of four states namely S- susceptible, E-exposed/infected, I-infectious and R-recovered/immune. We further allow for the possibility that individuals become infectious but the disease remains undiagnosed or progresses asymptomatically (we do not further discriminate between the causes of non-detection; it may be due to misdiagnosis or the absence of symptoms). A schematic representation of virus progression in the human population is presented in Figure S24.


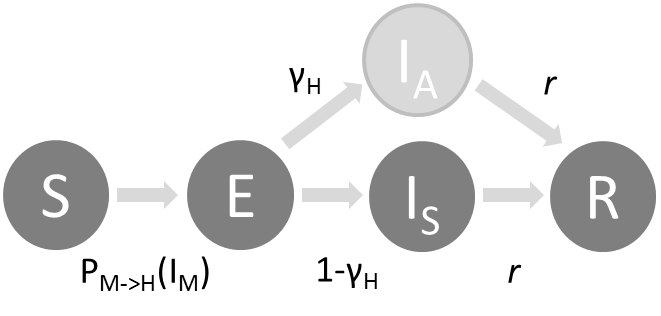


**Figure S24: Human infection states. Susceptible humans are infected with a probability depending on the number of infectious mosquitoes in the same cell.** The intrinsic incubation times and the durations of infection are γ distributed, based on the assumption that early progression to the next state is relatively rare.

Human individuals progress from the susceptible state (S) to the infected state (E) by being successfully infected from an infectious mosquito bite. The probability that such a bite is received depends on the number of infectious mosquitoes (I_M_) and the total number of mosquitoes (N_M_) that are co-located in the same cell of the modelled area. Each adult female mosquito (mosquito life cycle states A1 and A2) in assumed to bite humans at a certain frequency *ω* (the *biting rate*). We use estimates for the biting frequency of *Aedes aegypti* in Cairns that have been published previously. . We assume a constant biting rate in each simulation cycle; while *Aedes aegypti* has been observed to have a temperature dependent threshold of 15°C below which they are inactive [42], the climate in Cairns is such that activity can be assumed to be constant. We note that in other climatic settings vector biting activity could be modelled as a function of temperature. We do not explicitly account for interrupted feeds or repeated biting but assume that not every infectious bite leads to the successful establishment of human infection. Therefore the biting rate is multiplied with a ‘mosquito to human transmissibility’ which we call *a_1_*. The exact value for *a_1_* is unknown and published estimates are highly variable. We therefore adjust *a_1_* to calibrate the transmission model using the dengue outbreak data. The resulting probability that a given individual in a cell is infected at any time step of the simulation is given by Equation S9.

$P_{M\to H}=\frac{I_{M}\omega a_{1}}{N_{M}}$ (S9)

Whether a human is infected or not is dependent on the outcome of a binomial trial with the success probability *P_M->H_*. The variables *I_M_* and *N_m_* vary on each time step based on mosquito population and flight dynamics.

Once a human individual is infected, the virus requires a certain intrinsic incubation time (*γ_H_*) to replicate enough to cause the human to be classified as infectious. We assume that the duration of the intrinsic incubation periods is *gamma* distributed, i.e., it is unlikely that individuals become infectious before a certain threshold time has occurred.

In the model, the intrinsic incubation period is assigned to each individual at the time of infection, by sampling from a *gamma* distribution. The individual's intrinsic incubation period is recorded, and the transition from *E* to *I* is made in a later simulation cycle when this period has elapsed.

We use the *gamma* distribution parameters *ν/τ*=16 and *β_0_*=1.78 derived by Chan et al. 2012 from a literature review of incubation periods of dengue viruses.

When the intrinsic incubation time has passed, whether an individual continues the process on the symptomatic (*I_S_*) or the asymptomatic (*I_A_*) route is based on a binomial trial. We assume that 30-60% of all infections are asymptomatic. Infectious individuals are capable of transmitting the virus back to the susceptible mosquito population.

We assume that humans stay infectious for an average of 5-6 days. Infectious periods are also *gamma* distributed with the parameters *ν/τ*=6 and *β_0_*=1.15.

Once a human individual has reached the recovered/immune state (*R*), the same individual cannot be reinfected as we do not consider multiple co-circulating serotypes of dengue virus in the present model.

### **Infection and virus incubation for mosquitoes**

We consider both *A1* and *A2* stage mosquitoes to bite and transmit dengue virus. The infection process in the mosquitoes does not include a recovered/immune state as we assume that mosquitoes remain infected until they die. The resulting schematic representation of virus progression in the mosquito is shown in Figure S25.


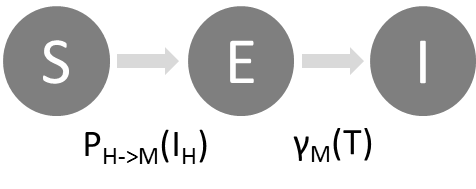


**Figure S25: Mosquito infection states.** Susceptible mosquitoes are infected with a probability based on the number of infectious humans in the same cell. The extrinsic incubation time is dependent on the temperature.

Similar to the transmission from mosquito to human, the reverse process (transmission from human to mosquito) is dependent on the number of infected humans (*I_H_*) and the number of total humans (*N_H_*) co-located with the mosquitoes in a cell, the mosquito biting rate ω and a *human to mosquito transmissibility* probability *a_2_* <1 to account for the fact that not every feed on an infectious human will lead to the individual mosquito becoming infected. The exact value of *a_2_* is unknown and published estimates vary considerably. We therefore let *a_2_* vary within the published ranges to calibrate the model using dengue outbreak data. Whether or not a mosquito becomes infected on a specific time step then depends on the outcome of a binomial trial with success probability *P_H->M_* as displayed in Equation 2

$P_{H\to M}=\frac{I_{H}\omega a_{2}}{N_{H}}$ (S10)

Once an individual adult mosquito is infected it depends on the temperature as to how long viral replication will take until infectious viral levels are reached. We assume that this extrinsic incubation period is *log normally* distributed for a given temperature as described by Chan et al. 2012. Since in their review article, Chan et al do not present the temperature specific parameter values of these log normal distributions, we used Figure 2D in to obtain estimates of log normal distribution means and standard deviations through a fitting process. The resulting temperature dependence of the extrinsic incubation periods is displayed in Figure S26.

In the model, the extrinsic incubation period of an individual mosquito is determined by sampling the log normal distribution for the temperature on the day of infection. We do not account for changing temperature once the extrinsic incubation period is assigned since we assume that mean daily temperatures do not change quickly relative to the extrinsic incubation period (which is of the order of 2-40 days).


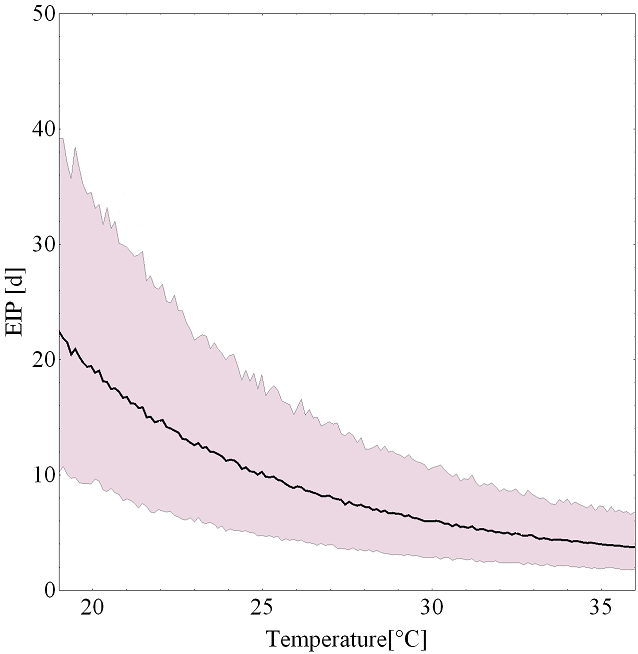


**Figure S26: Mean extrinsic incubation period dependence on temperature based on .**

If the mosquito survives the extrinsic incubation period, it enters the infectious state. It remains in the infectious state until it dies. We model mosquito life span based on a hazard function as detailed in the mosquito population dynamics model description.

In the present study we assume that dengue infection in *Aedes* *aegypti* does not impact on their population dynamics (i.e. does not affect lifespan or fecundity) and that there is no significant vertical transmission.

## Dengue Control Model

### **Tools for vector control**

The model accounts for conventional means of vector control that are applied in Cairns to control dengue spread during outbreaks. These include *i)* within-house residual spraying (IRS) and the treatment of yards and gardens with larvicide of case properties and those immediately adjacent to it and *ii)* the use of lethal ovitraps to kill egg-laying adults in extended areas around case properties. The control model is represented schematically in Figure S27.


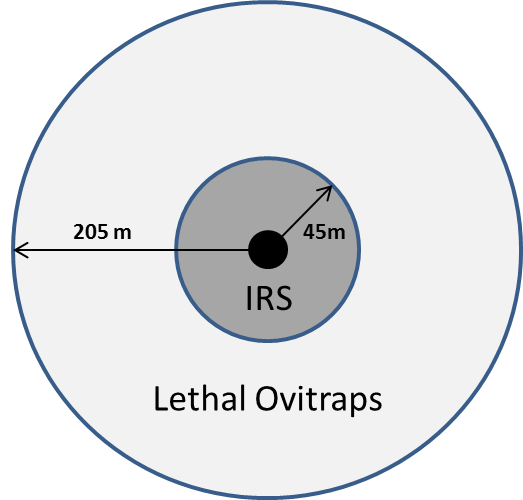


**Figure S27: For any reported/symptomatic dengue case, two control mechanisms are invoked**. The first is highly effective in-house residual spraying and larviciding in the case cell (black dot) and the cells adjacent to the case cell (dark grey). Lethal ovitraps are placed in a wider radius around the case cell.

We assume that IRS and larviciding in the immediate vicinity of the dengue case is highly effective, killing 90% of all adult and immature mosquito stages present in the case cell and the 8 surrounding cells immediately. The effect remains active for 6 weeks, so that 90% of mosquitoes entering the sprayed cell die immediately. This effectively means that the IRS treated cell will have no mosquitoes after a maximum of 2 days (99% of mosquitoes in the IRS cells will have died) and that, due to mosquito flight into the treated cells, surrounding cells will also be depleted of mosquitoes.

Lethal ovitraps have been shown to be an effective tool to control *Aedes aegypti.* Their deployment is rapid, and the biodegradable traps decompose after several weeks so that they do not have to be collected again . We assume that ovitraps will be deployed at a certain rate per day in the cells within a 205 m radius of a case. There are approximately 134 cells within the 205 m radius. Ovitraps kill approximately 20% of adult mosquitoes per cell so that the cells will be depleted of adult mosquitoes after 3-6 days. This effect is countered by the emergence of new mosquitoes, as the lethal ovitraps only target the adult stage.

### **Resource constraints**

From the above, it can be seen that even with a few (e.g. 10) new dengue cases on any given day may, would require IRS and larvicide treatment of up to 90 cells (81x10^3^ square meters) and more than 1250 cells being treated with ovitraps (approximately 1.1 square kilometres).

We assume that there is a limitation to the IRS and ovitrap coverage the can be achieved by the dengue control teams in on any day. If the required number of cells to be treated exceeds the maximum number treatable per day, the cells that are left untreated are carried over to the next day.

Our parameter choices for control are in line with those outlined in the current dengue response plan of Queensland Health. However, one of the capabilities of our model is to allow an analysis of alternative control strategies. Table S3 outlines all parameters used in the control part of the model.


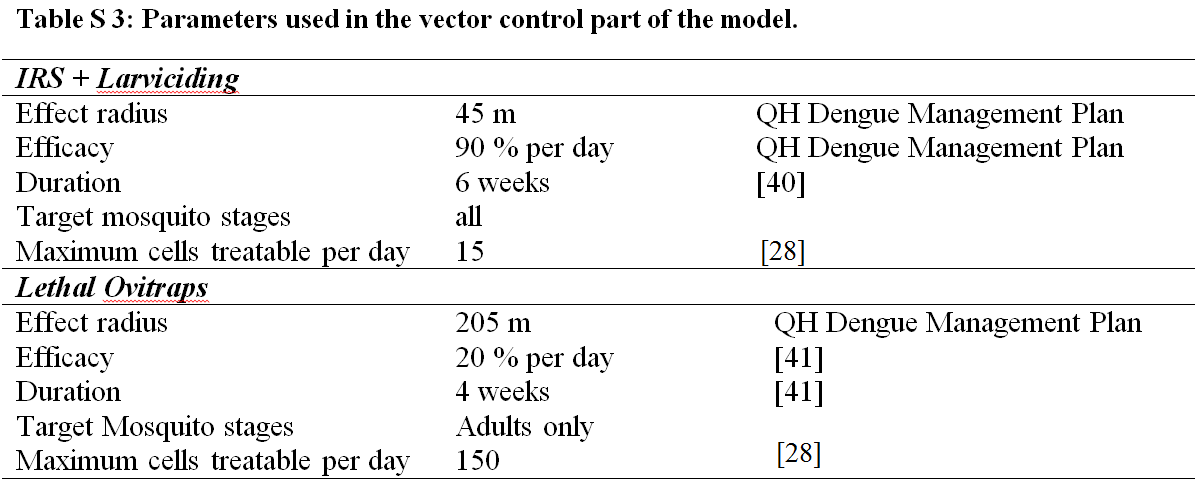


An example of these control mechanisms on the mosquito population is illustrated in Figure S28.


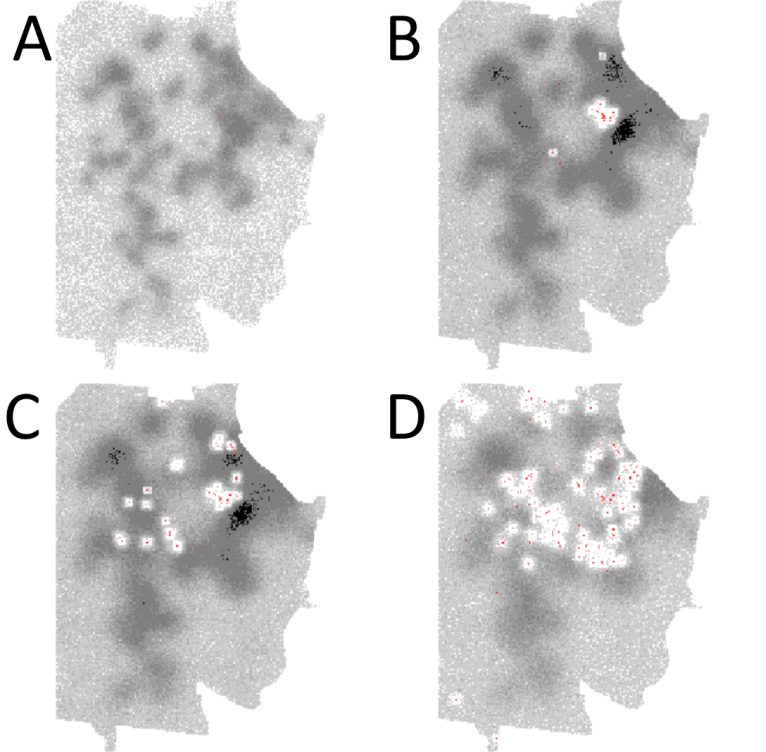


**Figure S28: Example of the effect of control on mosquito density during a simulated dengue outbreak.** Depending on the zones that are set for IRS/Larviciding, placement of lethal ovitraps and the settings for response time and resource limitation, each reported dengue case will invoke a response. The sequence of images (A-D) shows a simulated outbreak (based on the 2003 outbreak). The red markers indicated dengue case locations. The grey layer indicates local mosquito density in each model cell (white=0 mosquitoes, light grey=1-10 mosquitoes, dark grey=10-50 mosquitoes, black=50+ mosquitoes per cell). Since the outbreak occurred during a time when the seasonal mosquito density was still rising, the background mosquito density is lower in Panel A than in the other Panels. Videos of entire simulation sequences are supplied as additional files (Additional File 2 and Additional File 3).

The model may readily be extended to incorporate other intervention or prophylactic strategies targeted at limiting dengue epidemics, such as vaccination.

## List of necessary data

A list of necessary data required for the Cairns model is presented in Table S4.

Table S 4: A list of data used in the Cairns model

| **Datasets used in Cairns model** | **Brief description of datasets** |
| --- | --- |
| Census Data | Age distribution of individuals, household size and household age distribution. Detailed information is presented in Additional File 1 (page S4 – S6). |
| Cadastral Data | Cadastral data that specifies each location in Cairns geographically and is assigned one of 5 types: residential, commercial, industrial, educational or parkland. Households were only assigned to residential properties. Detailed information is presented in Additional File 1 (page S5 – S6). |
| Vegetation remote sensing Data | Satellite imaging and demographic data used to derive spatial information about mosquito habitats. Detailed information is presented in Additional File 1 (page S16 – S17). |
| Cairns mosquito trapping Data | The average number of trapped mosquitoes per trap per day in Cairns. Detailed information is presented in Additional File 1 (page S18 – S19). |
| Cairns dengue outbreak case Data | Spatially distributed Cairns dengue outbreak case data for 2003 and 2008/2009 outbreaks from Tropical Population Health Unit (TPHU) of Queensland Health. Detailed information is presented in the revised manuscript on page 7, 17 and 20. |

## References

1. Halder N, Kelso JK, Milne GJ: **Developing guidelines for school closure interventions to be used during a future influenza pandemic**. BMC Infect Dis 2010, 10: 221.

2. Halder N, Kelso JK, Milne GJ: **Analysis of the effectiveness of interventions used during the 2009 A/H1N1 influenza pandemic**. BMC Public Health 2010, 10: 168.

3. Milne GJ, Kelso JK, Kelly HA, Huband ST, McVernon J: **A small community model for the transmission of infectious diseases: comparison of school closure as an intervention in individual-based models of an influenza pandemic**. PLoS One 2008, 3: e4005.

4. Russell RC, Webb CE, Williams CR, Ritchie SA: **Mark-release-recapture study to measure dispersal of the mosquito Aedes aegypti in Cairns, Queensland, Australia**. Med Vet Entomol 2005, 19: 451-457.

5. Jones MDR: **The Programming of Circadian Flight-Activity in Relation to Mating and the Gonotrophic Cycle in the Mosquito, Aedes-Aegypti**. Physiological Entomology 1981, 6: 307-313.

6. May R, RM A: **Infectious diseases of humans: dynamics and control**. Oxford: Oxford University Press 1991.

7. Stoddard ST, Forshey BM, Morrison AC, Paz-Soldan VA, Vazquez-Prokopec GM, et al. **House-to-house human movement drives dengue virus transmission**. Proc Natl Acad Sci U S A 2013, 110: 994-999.

8. Vazquez-Prokopec GM, Stoddard ST, Paz-Soldan V, Morrison AC, Elder JP, et al. **Usefulness of commercially available GPS data-loggers for tracking human movement and exposure to dengue virus**. Int J Health Geogr 2009, 8: 68.

9. Mossong J, Hens N, Jit M, Beutels P, Auranen K, et al. **Social contacts and mixing patterns relevant to the spread of infectious diseases**. PLoS Med 2008, 5: e74.

10. Noulas A, Scellato S, Lambiotte R, Pontil M, Mascolo C: **A tale of many cities: universal patterns in human urban mobility**. PLoS One 2012, 7: e37027.

11. Otero M, Schweigmann N, Solari HG: **A stochastic spatial dynamical model for Aedes aegypti**. Bull Math Biol 2008, 70: 1297-1325.

12. Otero M, Solari HG, Schweigmann N: **A stochastic population dynamics model for Aedes aegypti: formulation and application to a city with temperate climate**. Bull Math Biol 2006, 68: 1945-1974.

13. Legros M, Magori K, Morrison AC, Xu C, Scott TW, et al. **Evaluation of location-specific predictions by a detailed simulation model of Aedes aegypti populations**. PLoS One 2011, 6: e22701.

14. Ellis AM, Garcia AJ, Focks DA, Morrison AC, Scott TW: **Parameterization and sensitivity analysis of a complex simulation model for mosquito population dynamics, dengue transmission, and their control**. Am J Trop Med Hyg 2011, 85: 257-264.

15. Focks DA, Haile DG, Daniels E, Mount GA: **Dynamic life table model for Aedes aegypti (diptera: Culicidae): simulation results and validation**. J Med Entomol 1993, 30: 1018-1028.

16. Focks DA, Haile DG, Daniels E, Mount GA: **Dynamic life table model for Aedes aegypti (Diptera: Culicidae): analysis of the literature and model development**. J Med Entomol 1993, 30: 1003-1017.

17. Focks DA, Daniels E, Haile DG, Keesling JE: **A simulation model of the epidemiology of urban dengue fever: literature analysis, model development, preliminary validation, and samples of simulation results**. Am J Trop Med Hyg 1995, 53: 489-506.

18. Sharpe PJ, DeMichele DW: **Reaction kinetics of poikilotherm development**. J Theor Biol 1977, 64: 649-670.

19. Schoolfield RM, Sharpe PJ, Magnuson CE: **Non-linear regression of biological temperature-dependent rate models based on absolute reaction-rate theory**. J Theor Biol 1981, 88: 719-731.

20. Chao DL, Halstead SB, Halloran ME, Longini IM, Jr.: **Controlling dengue with vaccines in Thailand**. PLoS Negl Trop Dis 2012, 6: e1876.

21. Styer LM, Carey JR, Wang JL, Scott TW: **Mosquitoes do senesce: departure from the paradigm of constant mortality**. Am J Trop Med Hyg 2007, 76: 111-117.

22. Williams CR, Johnson PH, Long SA, Rapley LP, Ritchie SA: **Rapid estimation of Aedes aegypti population size using simulation modeling, with a novel approach to calibration and field validation**. J Med Entomol 2008, 45: 1173-1179.

23. de Boer RJ: **Modeling Population Dynamics: a Graphical Approach**. University of Utrecht, 2013.

24. Vezzani D, Velazquez SM, Soto S, Schweigmann NJ: **Environmental characteristics of the cemeteries of Buenos Aires City (Argentina) and infestation levels of Aedes aegypti (Diptera: Culicidae)**. Mem Inst Oswaldo Cruz 2001, 96: 467-471.

25. Hanna JN, Ritchie SA, Merritt AD, van den Hurk AF, Phillips DA, et al. (1998) **Two contiguous outbreaks of dengue type 2 in north Queensland**. Med J Aust 1998, 168: 221-225.

26. Williams CR, Johnson PH, Ball TS, Ritchie SA: **Productivity and population density estimates of the dengue vector mosquito Aedes aegypti (Stegomyia aegypti) in Australia**. Med Vet Entomol 2013, 27: 313-322.

27. Anderson R, May R: **Infectious diseases of humans**. Oxford: Oxford University Press 1991. 768 p.

28. Williams CR, Long SA, Russell RC and Ritchie SA: **Field efficacy of the BG-Sentinel compared with CDC Backpack Aspirators and CO2-baited EVS traps for collection of adult *Aedes aegypti* in Cairns, Queensland, Australia**. Journal of the American Mosquito Control Association 2006, 22(2), 296-300.

29. Nevai AL, Soewono E: (2013) **A model for the spatial transmission of dengue with daily movement between villages and a city**. Math Med Biol 2013.

30. Otero M, Solari HG: **Stochastic eco-epidemiological model of dengue disease transmission by Aedes aegypti mosquito**. Math Biosci 2010, 223: 32-46.

31. Teixeira TR, Cruz OG: **Spatial modeling of dengue and socio-environmental indicators in the city of Rio de Janeiro, Brazil**. Cad Saude Publica 2011, 27: 591-602.

32. Canyon DV, Hii JL, Muller R: **Effect of diet on biting, oviposition, and survival of Aedes aegypti (Diptera: Culicidae)**. J Med Entomol 1999, 36: 301-308.

33. Andraud M, Hens N, Marais C, Beutels P: **Dynamic epidemiological models for dengue transmission: a systematic review of structural approaches**. PLoS One 2012, 7: e49085.

34. Wearing HJ, Rohani P, Keeling MJ: **Appropriate models for the management of infectious diseases**. PLoS Med 2005, 2: e174.

35. Chan M, Johansson MA: **The incubation periods of Dengue viruses**. PLoS One 2012, 7: e50972.

36. Endy TP, Chunsuttiwat S, Nisalak A, Libraty DH, Green S, et al. **Epidemiology of inapparent and symptomatic acute dengue virus infection: a prospective study of primary school children in Kamphaeng Phet, Thailand**. Am J Epidemiol 2002, 156: 40-51.

37. Adams B, Boots M: **How important is vertical transmission in mosquitoes for the persistence of dengue? Insights from a mathematical model**. Epidemics 2010, 2: 1-10.

38. Esu E, Lenhart A, Smith L, Horstick O: **Effectiveness of peridomestic space spraying with insecticide on dengue transmission; systematic review**. Tropical Medicine & International Health 2010, 15: 619-631.

39. **Queensland Dengue Mangement Plan 2010-2015**. Fortitude Valley: Queensland Health 2011.

40. Ritchie SA, Long S, Smith G, Pyke A, Knox TB: **Entomological investigations in a focus of dengue transmission in Cairns, Queensland, Australia, by using the sticky ovitraps.** J Med Entomol 2004, 41: 1-4.

41. Rapley LP, Johnson PH, Williams CR, Silcock RM, Larkman M, et al. **A lethal ovitrap-based mass trapping scheme for dengue control in Australia: II. Impact on populations of the mosquito Aedes aegypti**. Med Vet Entomol 2009, 23: 303-316.

42. Rowley WA, Graham CL: **Effect of temperature and relative humidity on flight ability of female *Aedes aegypti***. In Laboratory Studies of Mosquito Flight. Fort Detrick, Frederick, MD: Department of the Army; 1967.
