# Supplementary figures and images for: A spatial simulation model for dengue virus infection in urban areas
Source: BMC Infect Dis. 2014 Aug 20;14:447. doi: 10.1186/1471-2334-14-447 (PMC4152583; doi:10.1186/1471-2334-14-447)

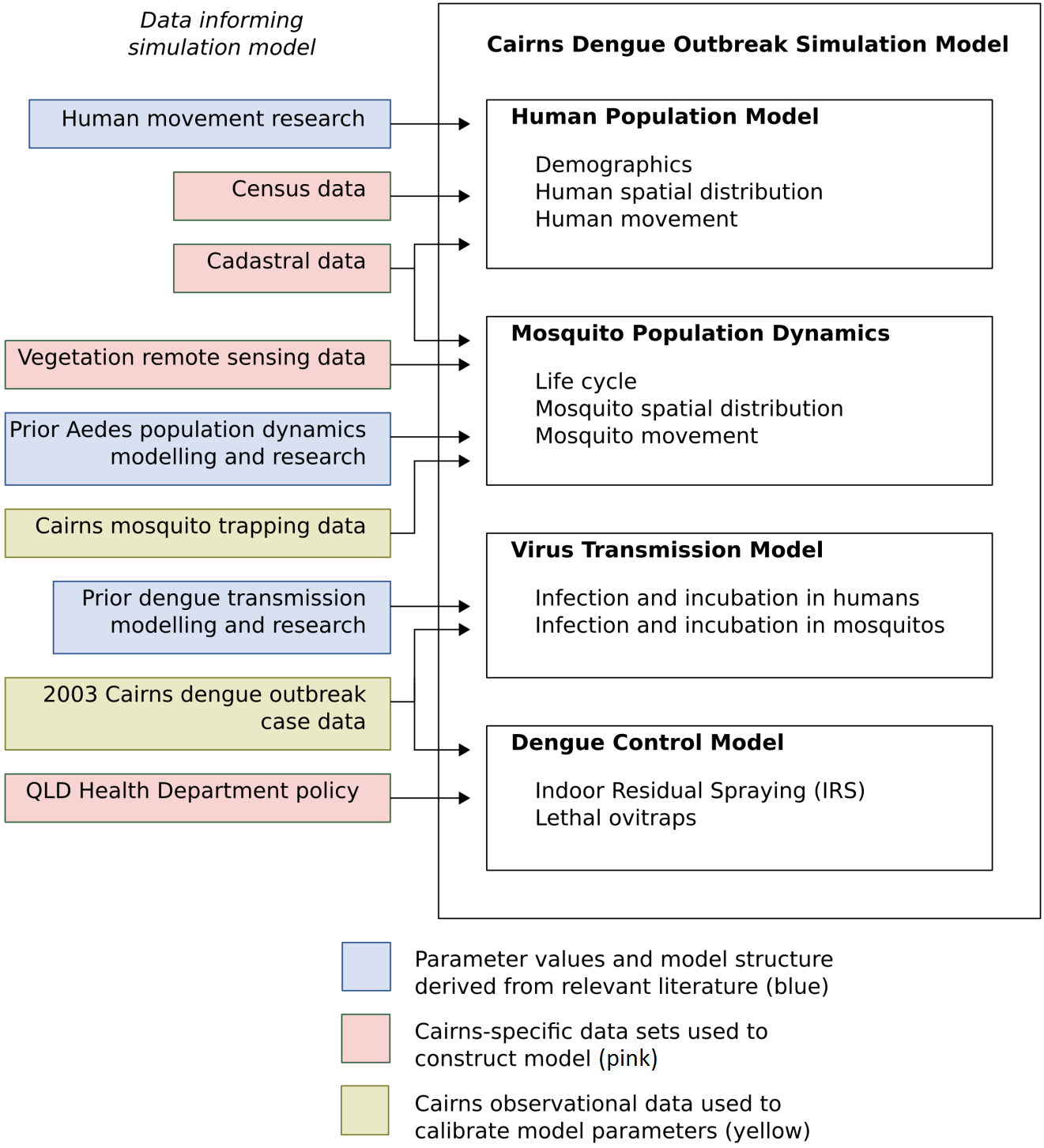

Supplement: Supplementary file 4 — Authors’ original file for figure 1 [file 12879_2014_3752_MOESM4_ESM.tif]

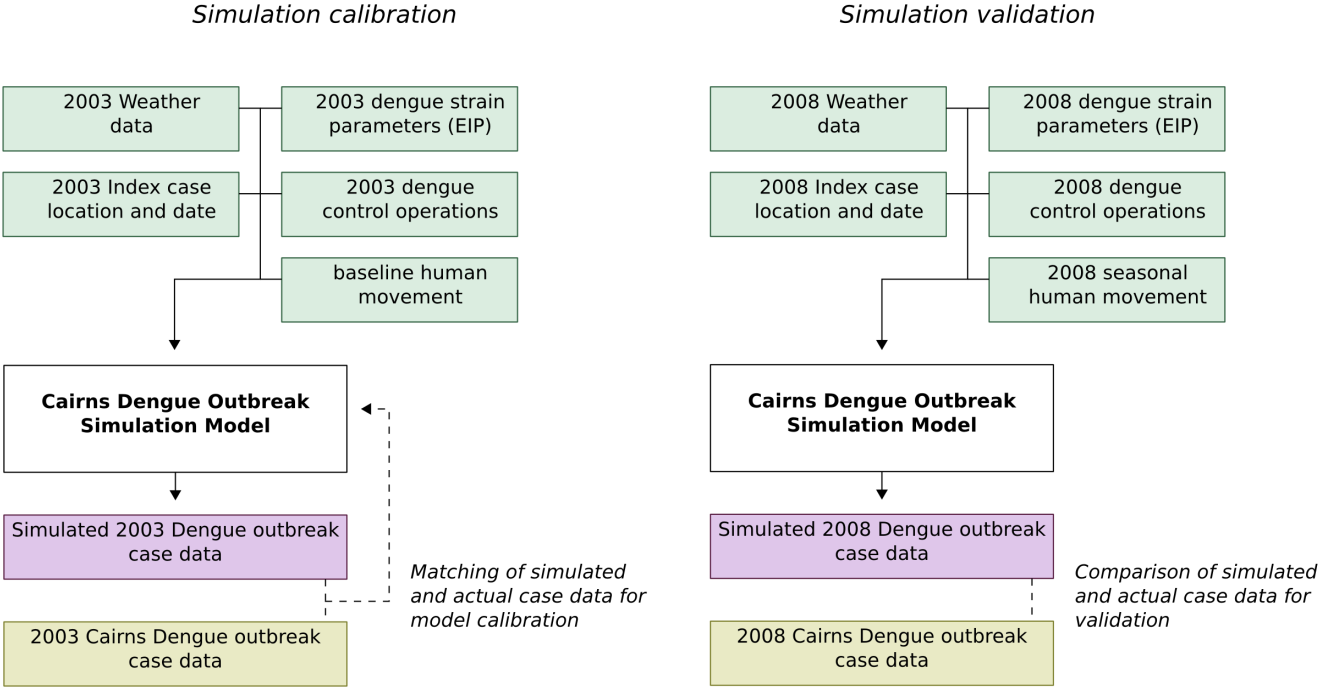

Supplement: Supplementary file 5 — Authors’ original file for figure 2 [file 12879_2014_3752_MOESM5_ESM.tif]

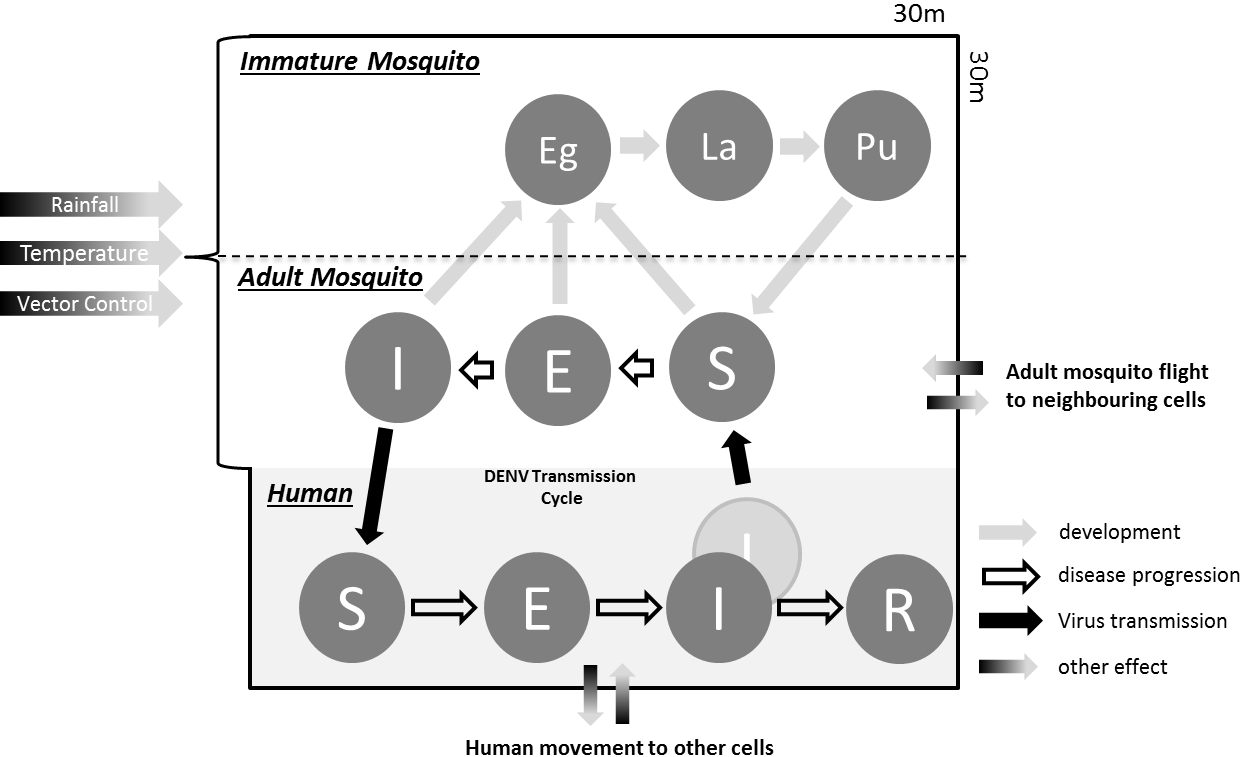

Supplement: Supplementary file 6 — Authors’ original file for figure 3 [file 12879_2014_3752_MOESM6_ESM.tif]

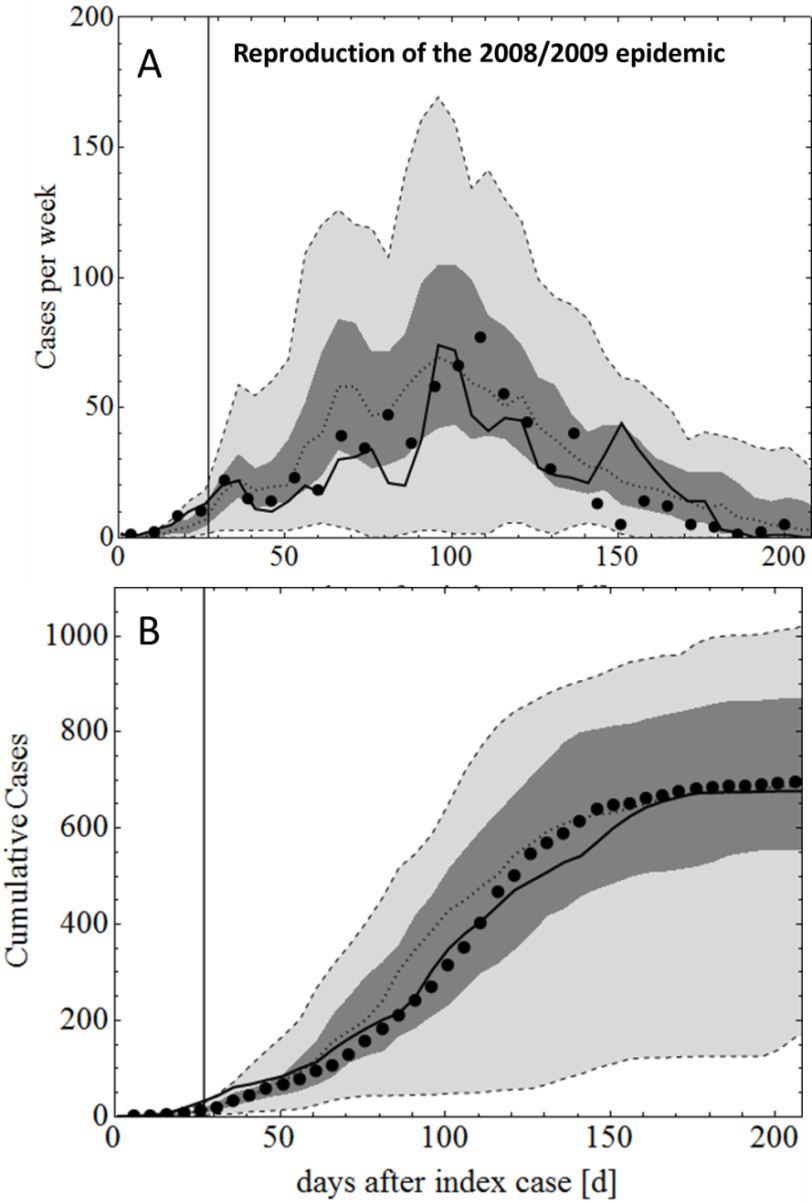

Supplement: Supplementary file 7 — Authors’ original file for figure 4 [file 12879_2014_3752_MOESM7_ESM.tif]

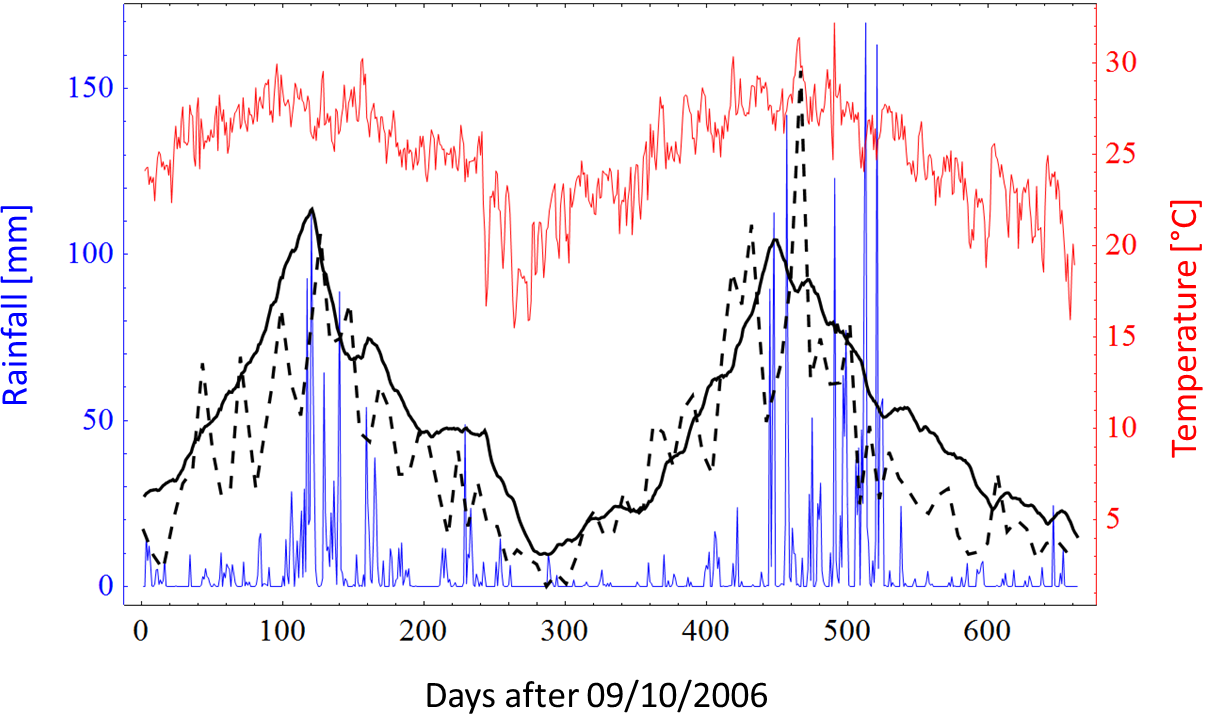

Supplement: Supplementary file 8 — Authors’ original file for figure 5 [file 12879_2014_3752_MOESM8_ESM.tif]

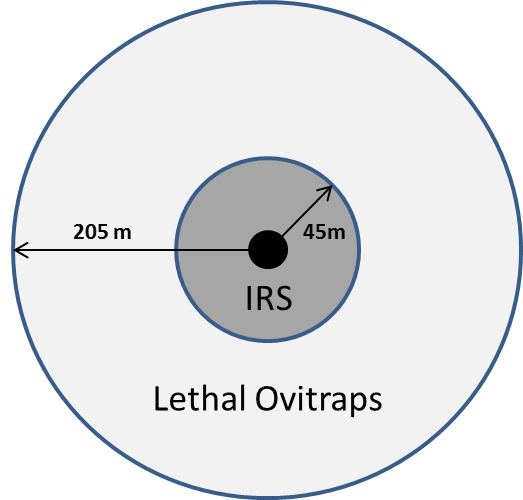

Supplement: Supplementary file 9 — Authors’ original file for figure 6 [file 12879_2014_3752_MOESM9_ESM.tif]

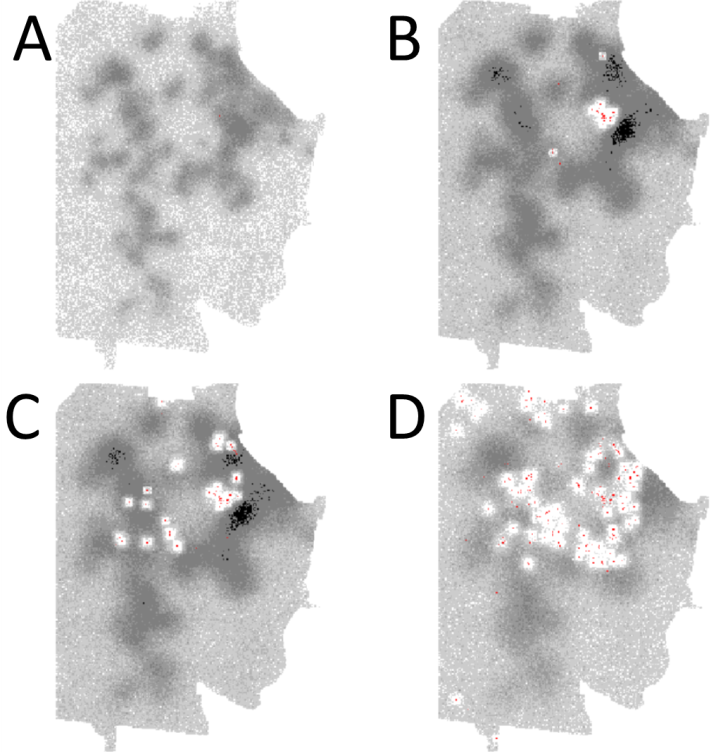

Supplement: Supplementary file 10 — Authors’ original file for figure 7 [file 12879_2014_3752_MOESM10_ESM.tif]

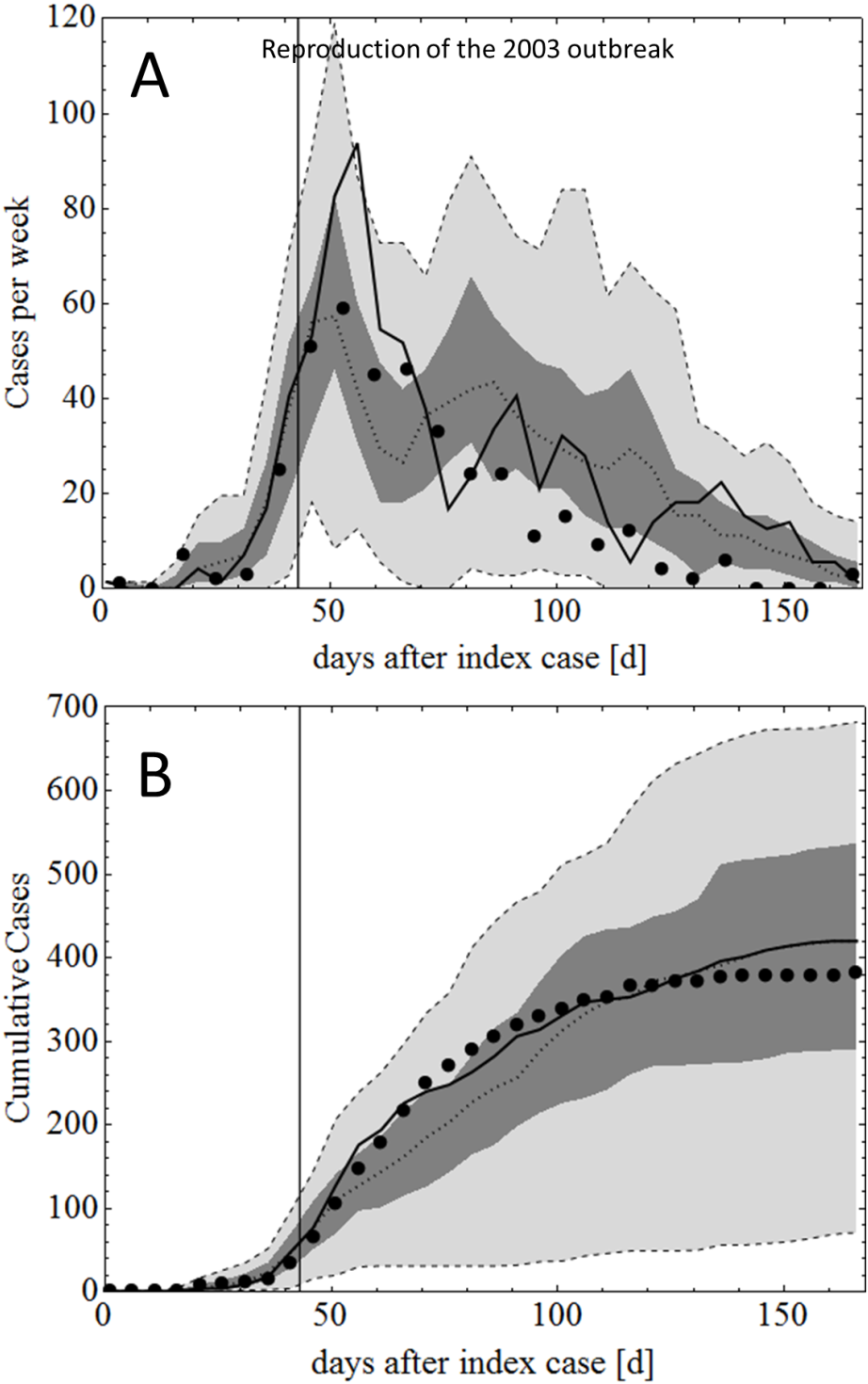

Supplement: Supplementary file 11 — Authors’ original file for figure 8 [file 12879_2014_3752_MOESM11_ESM.tif]

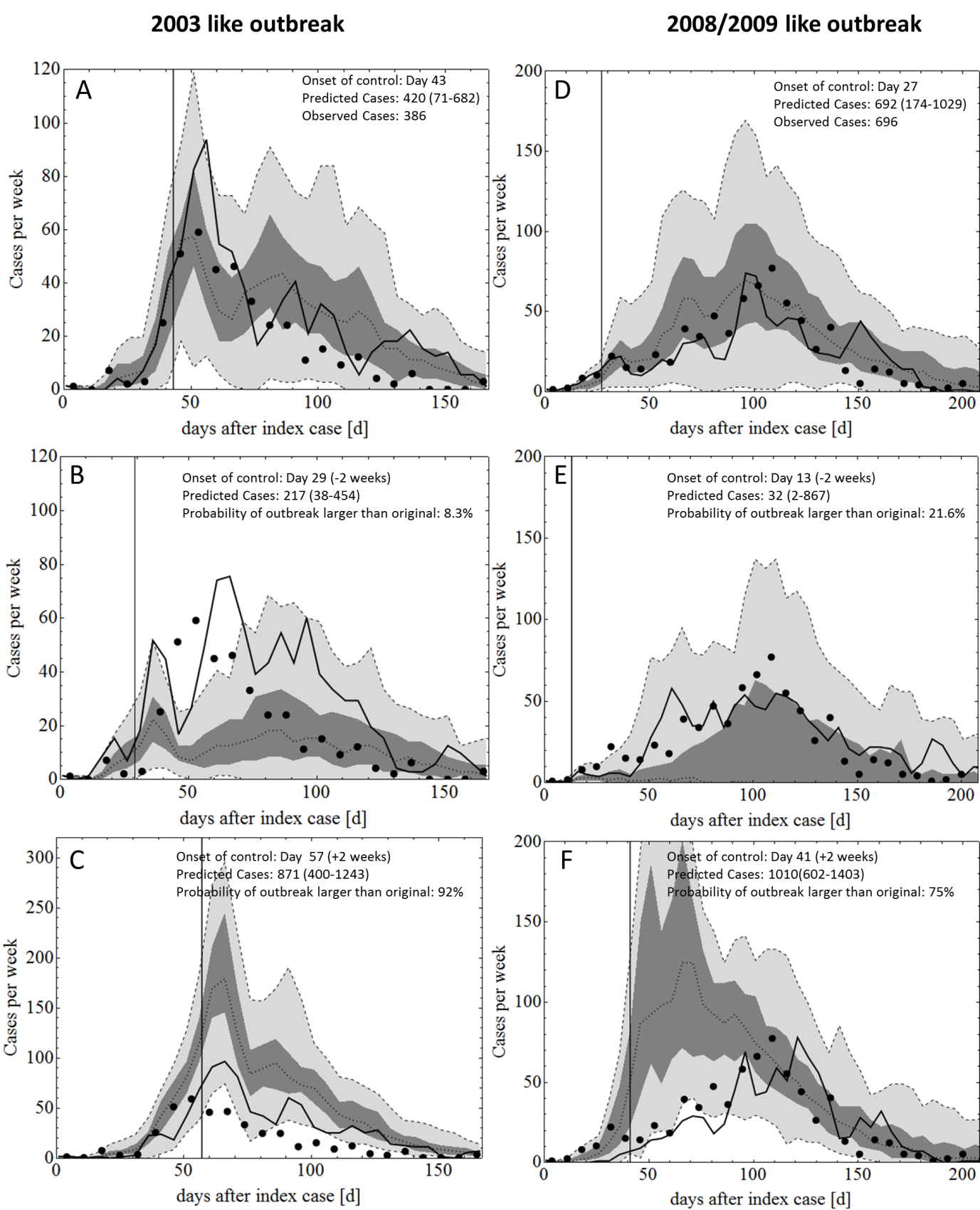

Supplement: Supplementary file 12 — Authors’ original file for figure 9 [file 12879_2014_3752_MOESM12_ESM.tif]

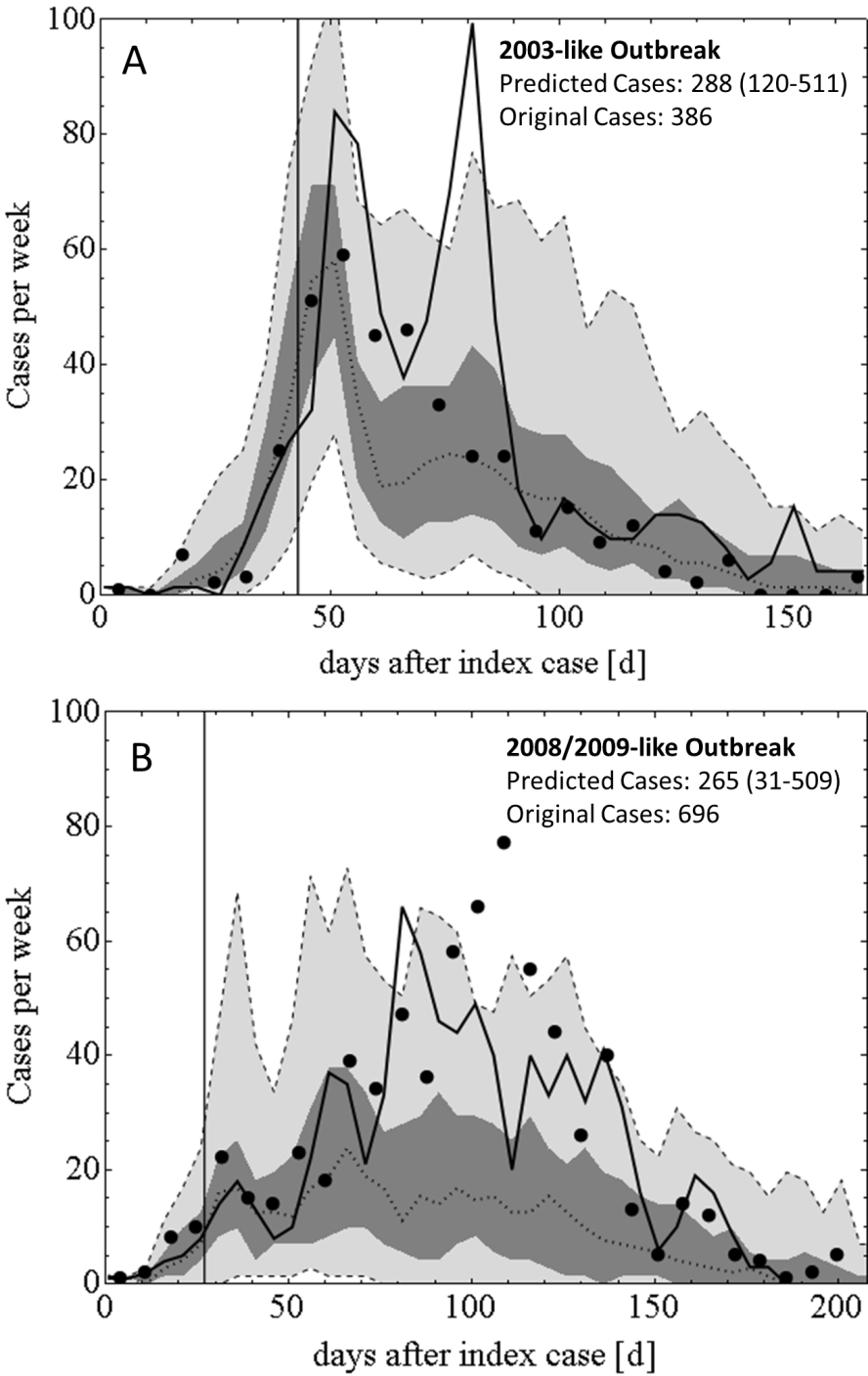

Supplement: Supplementary file 13 — Authors’ original file for figure 10 [file 12879_2014_3752_MOESM13_ESM.tif]
